# The kinase domain of TRPM7 interacts with PAK1 and regulates pancreatic cancer cell epithelial-to-mesenchymal transition

**DOI:** 10.1038/s41419-025-07665-2

**Published:** 2025-04-24

**Authors:** Julie Auwercx, Bernadette Neve, Alison Vanlaeys, Mathilde Fourgeaud, Ingrid Bourrin-Reynard, Mouloud Souidi, Sylvie Brassart-Pasco, Frédéric Hague, Stéphanie Guenin, Belinda Duchene, Laurent Gutierrez, Olivier Destaing, Isabelle Dhennin-Duthille, Isabelle Van Seuningen, Nicolas Jonckheere, Mathieu Gautier

**Affiliations:** 1 Université de Picardie Jules Verne, UR-UPJV 4667, Amiens, France; 2 Univ. Lille, CNRS, Inserm, CHU Lille, UMR9020-U1277—CANTHER—Cancer Heterogeneity Plasticity and Resistance to Therapies, Lille, France; 3 Institute for Advanced Biosciences, University Grenoble Alpes, INSERM U1209, CNRS UMR5309, site santé, Allée des Alpes, Grenoble, France; 4 Université de Reims Champagne-Ardenne, CNRS, MEDYC, Reims, France; 5 Université de Picardie Jules Verne, Centre de Ressources Régionales en Biologie Moléculaire (CRRBM), Amiens, France

**Keywords:** Cancer, Oncogenesis

## Abstract

Pancreatic ductal adenocarcinoma (PDAC) is the main and the deadliest form of pancreatic cancer. This is a major problem of public health since it will become the second leading cause of death by cancer in the next few years, mainly due to the lack of efficient therapies. Transient Receptor Potential Cation Channel Subfamily M Member 7 (TRPM7) protein, a cation channel fused with a serine/threonine kinase domain is overexpressed in PDAC and associated with a low survival. In this work, we aim to study the role of kinase domain on pancreatic cell fates by using a model of kinase domain deletion by CRISPR-Cas9. PANC-1 and MIA PaCa-2 PDAC cell lines were used and kinase domain was deleted by CRISPR-Cas9 strategy. Kinase domain deletion (ΔK) was validated by RT-qPCR and western blots. The effect of kinase domain deletion on channel function was studied by patch-clamp and Mn^2+^-quenching. The cell phenotype was studied by MTT and cell migration/invasion assays. Finally, the role of kinase domain was studied in vivo in xenografted mice. Here we show that TRPM7 kinase domain is required to maintain a mesenchymal phenotype in PDAC cells. We also demonstrated that TRPM7 and PAK1 interact in the same protein complexes. Moreover, TRPM7 kinase domain is required for carcinogenesis and cancer cell dissemination in vivo. Intriguingly, the role of TRPM7 kinase is cell specific and may depend on the KRAS oncogene mutation status. In conclusion, TRPM7 kinase domain is required to maintain a mesenchymal and aggressive phenotype in PDAC cells, and it could be a promising target against PDAC.

## Introduction

Pancreatic Ductal AdenoCarcinoma (PDAC) develops in the exocrine tissue and represents more than 90% of all pancreatic cancers [1]. In 2022, PDAC was the 12th cancer in terms of incidence and the 6th in terms of mortality worldwide [2]. Epidemiological projections estimate that PDAC will become the 2nd most common cause of cancer-related death in 2040 [3]. PDAC is a silent killer with a 5-year survival rate of almost 10% due to the resistance of cancer cells to chemotherapy and to their metastatic phenotype [1]. The metastatic process occurs during a mechanism called ‘metastatic cascade’ [4]. The first step of the metastatic cascade is the transition of epithelial toward mesenchymal phenotype (epithelial-to-mesenchymal transition or EMT) leading to enhanced cell migration and invasion. A better comprehension of molecular mechanisms involved in EMT and cell metastatic properties may help to propose new strategies to fight this cancer [5].

Ion channels are membrane proteins that have been shown to participate in numerous cancer mechanisms and are considered as promising therapeutic targets [6]. We have already highlighted the role of Transient Receptor Potential Cation Channel Subfamily M member 7 (TRPM7) in PDAC cell migration and invasion [7–9]. TRPM7 is a dual-function protein composed by a divalent cation permeant channel (Ca^2+^, Mg^2+^ and Zn^2+^) fused with a kinase domain at its C-terminus [10, 11]. While the channel function is essential for Mg^2+^ absorption, Mg^2+^ cellular homeostasis and survival [12, 13], the kinase is involved in resistance to Mg deprivation but its physiological role is far from understood [14]. In breast cancer cells, the TRPM7 kinase is involved in cell migration, metastasis formation and dissemination [15, 16]. Several signaling pathways could be regulated by TRPM7 kinase interaction with identified substrates but it is not clear how this could affect oncogenic signalization [17].

In this study, we aimed to assess the role of TRPM7 kinase domain in regulating PDAC cell metastatic properties and carcinogenesis in vivo by engineering an endogenous TRPM7 with a deletion of the C-terminal part containing the kinase domain.

## Results

### Endogenous TRPM7 kinase deletion had no effect on channel activity in PDAC cell lines

**Fig. 1:**
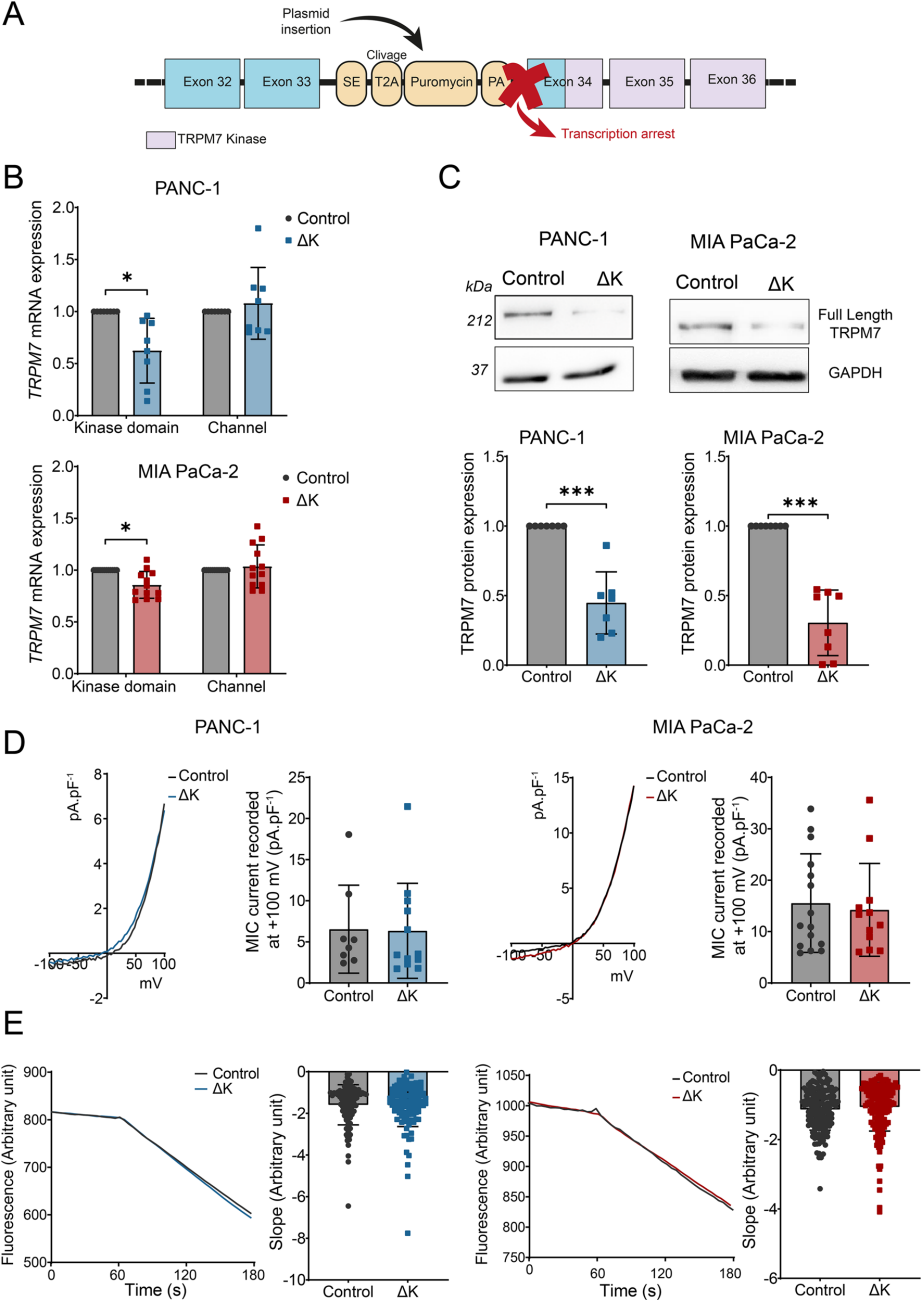
TRPM7 kinase domain deletion characterization in PDAC cell lines. **A** Schematic representation of CRISPR-Cas9 model deletion of TRPM7 kinase domain in PANC-1 and MIA PaCa-2 cell lines. **B** mRNA levels of *TRPM7* mRNA using primers targeting the kinase domain and the channel in PANC-1 (*n* = 8, *p* < 0.05) and MIA PaCa-2 (*n* = 12, *p* < 0.05) cells (Two-way ANOVA followed by Šidák’s *post-hoc* test). **C** Protein levels of full-length TRPM7 showing a decrease of ~55% in PANC-1 (*n* = 7, *p* < 0.001), and a decrease of ~70% in MIA PaCa-2 (*n* = 7, *p* < 0.001) using a TRPM7 antibody raised against the kinase domain (Mann–Whitney test). **D** Electrophysiological recordings of TRPM7 currents in PANC-1 cells (Control, n = 8; ΔK, n = 12) and MIA PaCa-2 cells (Control, *n* = 5; ΔK, *n* = 12) and quantification of MIC currents at +100 mv (Mann–Whitney test). **E** Constitutive entry of divalent cations estimated by manganese quench in PANC-1 (Control, *n* = 107; ΔK, *n* = 117) and MIA PaCa-2 (Control, *n* = 176; ΔK, *n* = 185) cells (Mann–Whitney test). Results are shown as mean ± SD. *p < 0.05; ***p < 0.001.

We aimed to study the role of TRPM7 kinase domain by using the CRIPSR-Cas9 strategy to delete the endogenous C-terminus part (containing the kinase domain) at the exon 34 of *TRPM7* gene (Fig. 1A), resulting in TRPM7 protein truncation after amino acid R1578, in PANC-1 and MIA PaCa-2 PDAC cell lines. The deletion of TRPM7 kinase domain (ΔK) has been validated by RT-qPCR using primers targeting the kinase domain ARNm or the channel ARNm sequence, respectively (Fig. 1B). The TRPM7 kinase domain deletion has been also validated by immunoblotting using antibody targeting specifically the TRPM7 kinase domain (ab245408 antibody from Abcam, which recognizes its epitope on aa residue 1800 in the kinase domain at the C-terminus), and thus detecting only the full-length protein. The deletion was not complete as we detected a full-length TRPM7 expression decrease of almost 55% in PANC-1, and a decrease of almost 70% in MIA PaCa-2 cells (Fig. 1C). The Magnesium-Inhibited Cation (MIC) currents were recorded by whole-cell patch-clamp as previously described [8, 18, 19] to assess the effect of the kinase deletion on the channel activity (Fig. 1D). The MIC current densities were not significantly different between the control and the ΔK cells suggesting that the kinase domain deletion had no effect on the channel activity. As TRPM7 has been shown to be involved in cation constitutive entry, we also used Mn^2+^-quench experiments to study the effect of TRPM7 kinase domain deletion on cellular Mn^2+^ influxes. The cation constitutive entry was similar between control and ΔK cells (Fig. 1E). Taken together, these results showed that our model of TRPM7 deletion did not affect the channel activity in PDAC cells suggesting that TRPM7 channel function is independent of the kinase domain in our cell models.

### TRPM7 kinase deletion modified MgATP sensitivity of MIC currents

**Fig. 2:**
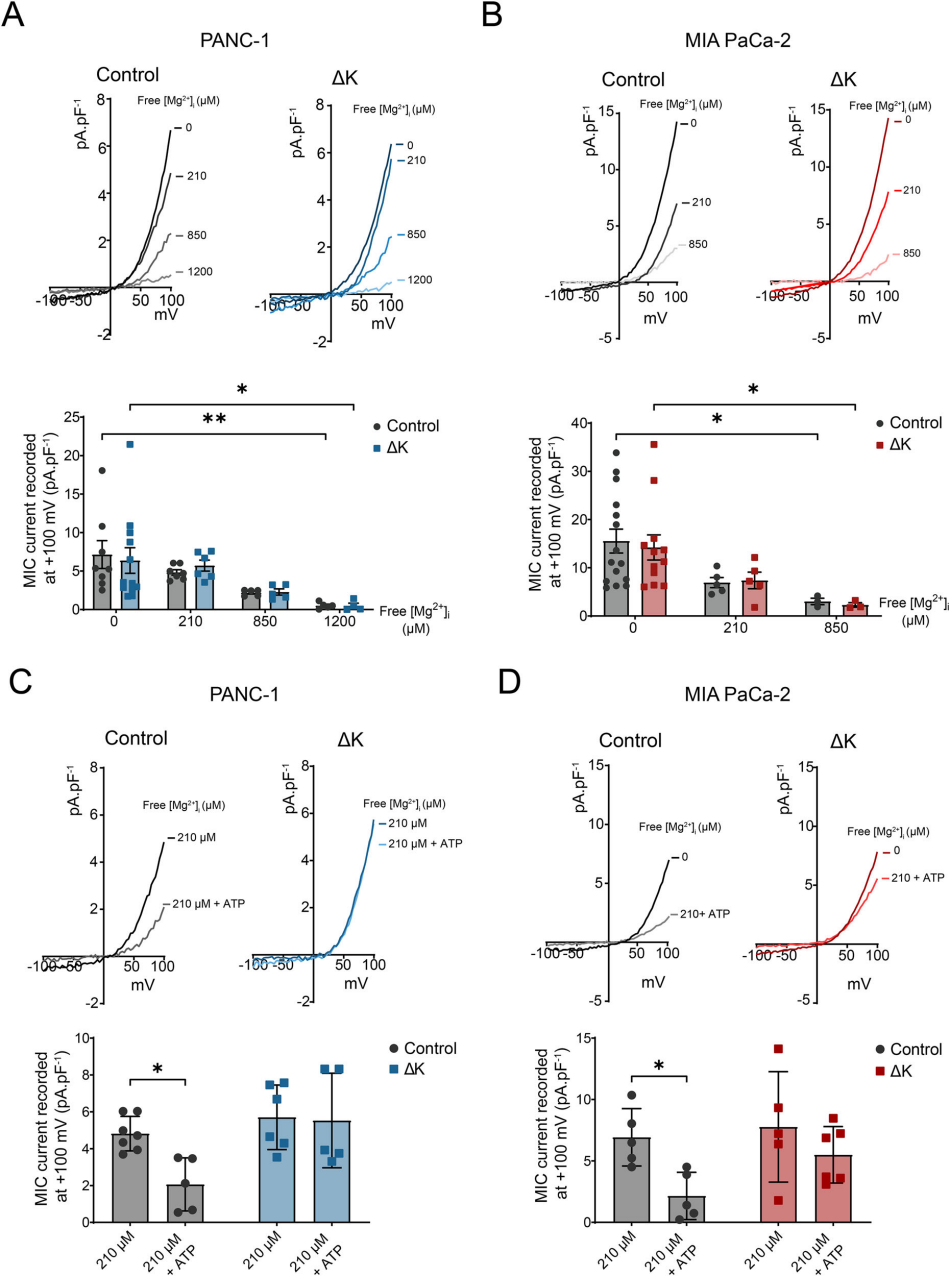
TRPM7 kinase domain deletion modifies MgATP sensitivity of MIC currents without effect on total current densities. **A** Electrophysiological recordings of TRPM7 currents in PANC-1 Control and ΔK cells with 0 µM (Control, *n* = 8; ΔK, *n* = 12), 210 µM (Control, *n* = 7; ΔK, *n* = 6), 850 µM (Control, *n* = 5; ΔK, *n* = 5) and 1200 µM (Control, *n* = 5; ΔK, *n* = 4) of Mg^2+^ in intrapipette solution and quantification of MIC currents at +100 mV (*p < 0.05; **p < 0.01, Two-way ANOVA followed by Šidák’s *post-hoc* test). **B** Electrophysiological recordings of TRPM7 currents in MIA PaCa-2 Control and ΔK cells with 0 µM (Control, *n* = 15; ΔK, *n* = 12), 210 µM (Control, *n* = 5; ΔK, *n* = 5) and 850 µM (Control, *n* = 3; ΔK, *n* = 3) of Mg^2+^ in intrapipette solution and quantification of MIC currents at +100 mV (*p < 0.05, Two-way ANOVA followed by Šidák’s *post-hoc* test). **C** Electrophysiological recordings of TRPM7 currents in PANC-1 control cells containing 210 µM free Mg^2+^ (*n* = 7) or containing 210 µM free Mg^2+^ and MgATP (*n* = 5; *p < 0.05) and in ΔK cells containing 210 µM free Mg^2+^ (*n* = 6) or containing 210 µM free Mg^2+^ and MgATP (*n* = 5). Below: quantification of MIC currents at +100 mV (Two-way ANOVA followed by Šidák’s *post-hoc* test). **D** Same experiments realized in MIA PaCa-2 control cells containing 210 µM free Mg^2+^ (*n* = 5) or containing 210 µM free Mg^2+^ and MgATP (*n* = 5; *p* < 0.05), and in ΔK cells containing 210 µM free Mg^2+^ (*n* = 5) or containing 210 µM free Mg^2+^ and MgATP (*n* = 6). Below: quantification of MIC currents at +100 mV (Two-way ANOVA followed by Šidák’s *post-hoc* test). Results are shown as mean ± SD. *p < 0.05; **p < 0.01.

One of the features of TRPM7 channel is its sensitivity to intracellular free Mg^2+^ and MgNTP [10, 11]. MIC Currents were significantly inhibited by intracellular free Mg^2+^ in both PDAC cell lines. Moreover, the sensitivity of MIC currents to intracellular free Mg^2+^ was not modified by the kinase domain deletion (Fig. 2A, B). Adding ATP in the intrapipette solution increased the sensitivity of MIC currents to intracellular free Mg^2+^ in control cells but we observed a loss of MIC current sensitivity to MgATP in ΔK cells (Fig. 2C, D). These results showed that the kinase domain deletion modified MgATP sensitivity of MIC currents.

### TRPM7 kinase deletion maintained epithelial phenotype in Panc-1 cells

**Fig. 3:**
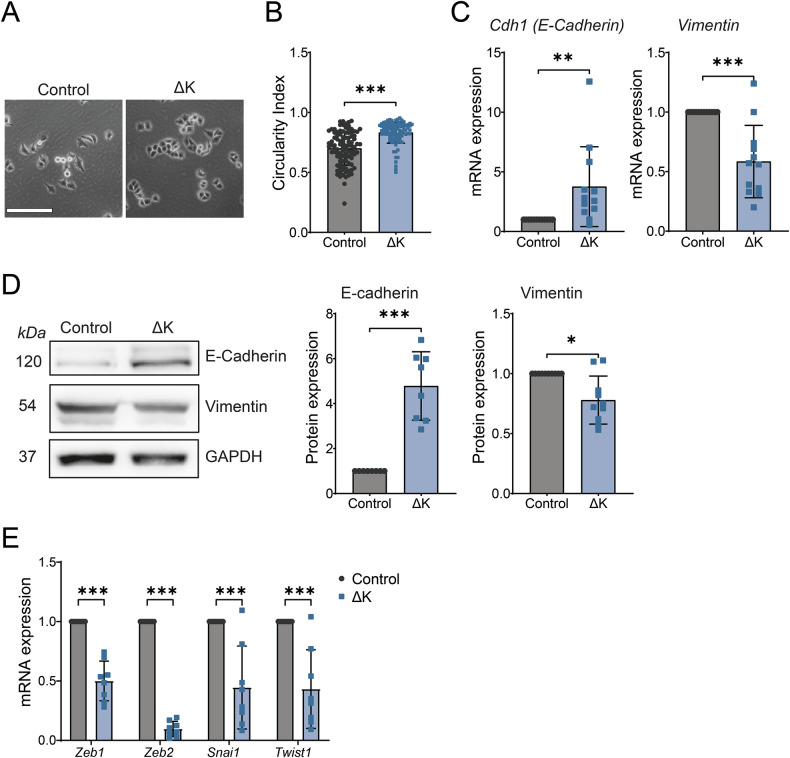
TRPM7 kinase domain deletion maintains epithelial phenotype in PANC-1 cells. **A** Representative morphology of PANC-1 Control and ΔK cells at ×100 magnification. Scale bar = 200 µm. **B** Circularity index calculated for PANC-1 Control (*n* = 98) and ΔK (*n* = 106) cells (*p* < 0.001, Mann–Whitney test). **C** mRNA expression of *CDH1* (*n* = 12, *p* < 0.01, Mann–Whitney test) and Vimentin (*n* = 12, *p* < 0.001, Mann–Whitney test) in PANC-1 Control and ΔK cells. **D** Protein expression of E-cadherin (*n* = 8, *p* < 0.001, Mann–Whitney test) and Vimentin (*n* = 8, *p* < 0.05, Mann–Whitney test) in PANC-1 Control and ΔK cells. **E** mRNA expression of EMT transcription factors *Zeb1, Zeb2, Snai1 and Twist1* (*n* = 8, *p* < 0.001, Two-way ANOVA followed by Šidák’s *post-hoc* test) in PANC-1 Control and ΔK cells. Results are shown as mean ± SD. *p < 0.05; **p < 0.01; ***p < 0.001.

The PANC-1 cell line was chosen for the rest of the study because it possesses the KRAS G12D mutation which is the most selected KRAS mutation detected in PDAC [20, 21]. Moreover, the KRAS G12D mutation is associated with worse overall survival in PDAC [22]. A change of cell shape was observed for ΔK cells compared to the control ones (Fig. 3A). The ΔK cells were more rounded than the control ones as attested by the calculated circularity index (Fig. 3B). This change of cell morphology was accompanied by an increase of both E-cadherin mRNA and protein expression in ΔK cells when compared to the control ones and a decrease of vimentin mRNA and protein expression (Fig. 3C, D). These data strongly suggest that the TRPM7 kinase domain is required for maintaining a mesenchymal phenotype in PANC-1 cells. To confirm this hypothesis, the expression epithelial-to-mesenchymal transition (EMT)-associated transcription factors was also studied by RT-qPCR. We showed that *Snai1*, *Twist1*, *Zeb1* and *Zeb2* mRNA expressions were decreased in ΔK cells compared to the control ones (Fig. 3E).

### TRPM7 kinase is required for PANC-1 cell migration and invasion

**Fig. 4:**
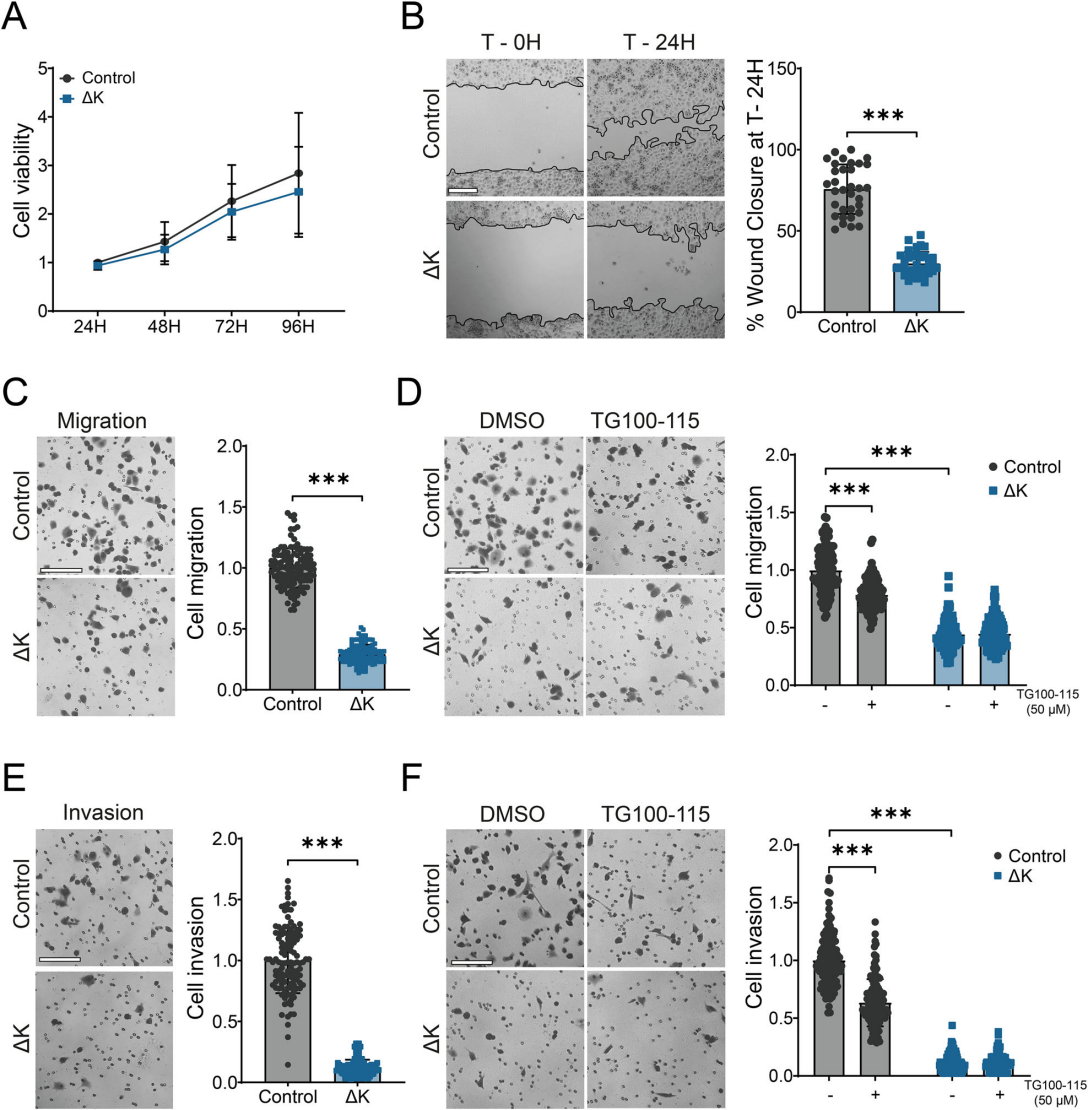
TRPM7 kinase domain is required for PANC-1 migration and invasion. **A** Cell viability of PANC-1 Control and ΔK cells assessed by MTT assay during 96 h (Two-way ANOVA). **B** Cell migration of PANC-1 Control and ΔK cells assessed by wound-healing assay (*n* = 3, *p* < 0.001, Student-*t* test). Photographs were taken at magnification x50 right after the wound (T–0H) and 24 h after (T–24H). **C** Cell migration of PANC-1 Control and ΔK evaluated in Boyden chambers (*n* = 3, *p* < 0.001, Mann–Whitney test). **D** Cell migration of PANC-1 Control and ΔK treated with TG100-115 (50 µM) for 24 h (*n* = 3, *p* < 0.001, Two-way ANOVA followed by Šidák’s *post-hoc* test). **E** Cell invasion of PANC-1 Control and ΔK evaluated in Boyden chambers coated with Matrigel (*n* = 3, *p* < 0.001, Mann–Whitney test). **F** Cell invasion of PANC-1 Control and Δ*K* treated with TG100–115 (50 µM) for 24 h (*n* = 3, *p* < 0.001, Two-way ANOVA followed by Šidák’s *post-hoc* test). All the scale bars are corresponding to 200 µm. Results are shown as mean ± SD. ***p < 0.001.

We previously showed that TRPM7 silencing decreased both pancreatic cancer cell migration and invasion [8, 9]. Here we assess more specifically the role of the TRPM7 kinase domain in these mechanisms. Firstly, we studied the cell viability by using MTT assays. The kinase domain deletion had no effect on cell viability (Fig. 4A). On the other hand, we observed a decrease of the directional migration (wound-healing assay, Fig. 4B) and the migration induced by chemoattraction (Boyden chamber assay, Fig. 4C). We used TG100-115 (50 µM) as a pharmacological blocker of TRPM7 kinase activity [23] and we showed a decrease of cell migration in control cells with no additive effect in ΔK cells (Fig. 4D). Similar results were observed for cell invasion in Matrigel-modified Boyden chambers (Fig. 4E, F). Taken together, our results showed that the TRPM7 kinase domain promoted a mesenchymal-like phenotype in PDAC cells leading to enhanced cell migration and invasion. Moreover, we confirmed the effect of TG100-115 on another PDAC cell line, AsPC-1, which harbors the same KRAS G12D mutation (Fig. S5).

### TRPM7 kinase regulated adhesion complex protein and small GTPase expressions

**Fig. 5:**
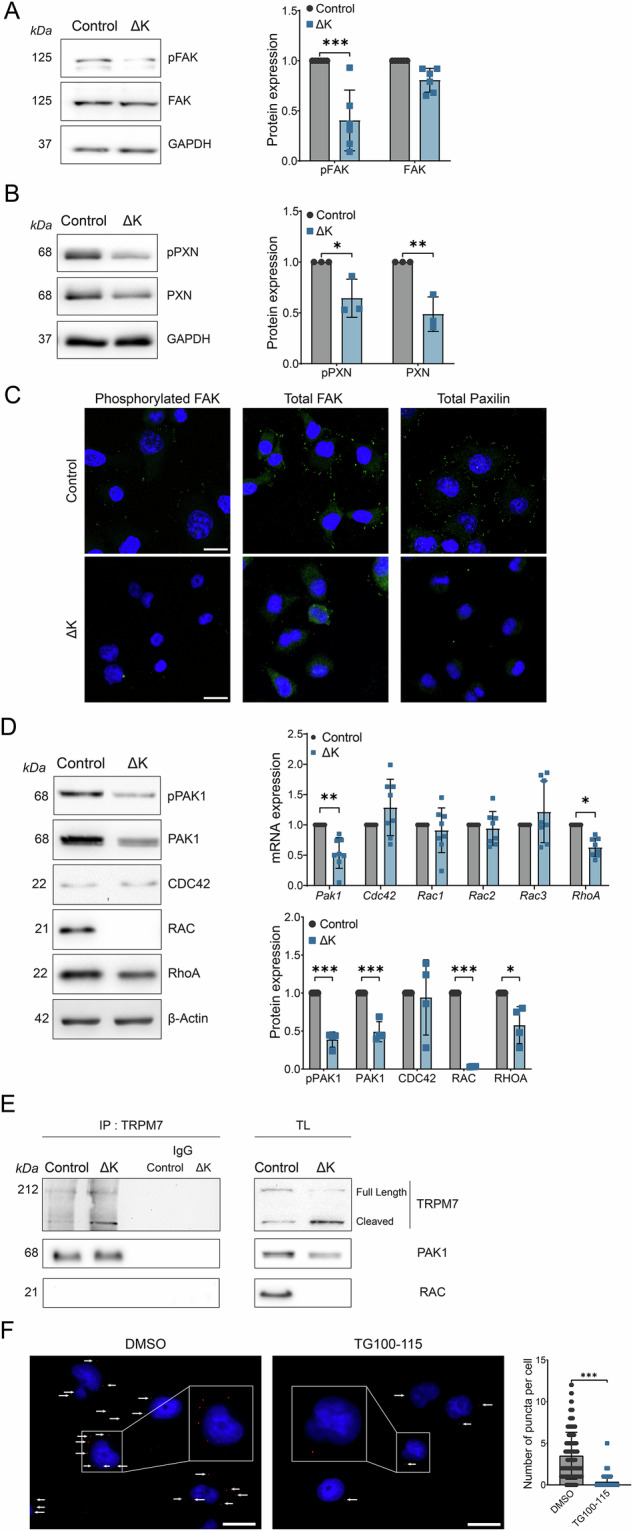
TRPM7 kinase domain interacts with PAK1. **A** Protein expression levels of pFAK and FAK in PANC-1 Control and ΔK cell lines (*n* = 6, *p* < 0.001, Two-way ANOVA followed by Šidák’s *post-hoc* test). **B** Protein expression levels of pPXN and PXN in PANC-1 Control and ΔK cell lines (*n* = 3, *p* < 0.05 and *p* < 0.01, Two-way ANOVA followed by Šidák’s *post-hoc* test). **C** Detection of focal adhesion complexes by immunofluorescence targeting pFAK, FAK and PXN. **D** Evaluation of the RhoGTPase family genes *Pak1*, *Cdc42*, *Rac1*, *Rac2*, *Rac3* and *RhoA* assessed by RT-qPCR (*n* = 8 *p* < 0.05 and *p* < 0.01, Two-way ANOVA followed by Šidák’s *post-hoc* test) and by Western-Blot (*n* = 4, *p* < 0.05, *p* < 0.01 and *p* < 0.001, Two-way ANOVA followed by Šidák’s *post-hoc* test). **E** Co-immunoprecipitation of TRPM7 with PAK1 and RAC proteins in PANC-1 Control and ΔK cells and their respective positive and negative controls. **F** Proximity Ligation assay of TRPM7 with PAK1 in PANC-1 cells incubated with DMSO (*n* = 162) or 50 µM TG100-115 (*n* = 177) for 24 h (Mann–Whitney test). Scale bar = 20 µm. Results are shown as mean ± SD. *p < 0.05; **p < 0.01; ***p < 0.001.

Cell migration and invasion mechanisms were due to balance between cell adhesion and detachment mechanisms as well as remodeling of myofilaments involving a cascade of intracellular signaling including small GTPases. Firstly, we assessed the expression of Focal Adhesion Kinase (FAK) protein involved in the cell adhesion complex and cell migration regulation. Phosphorylated FAK (pFAK) expression was decreased in ΔK cells compared to the control ones without effect on total FAK expression (Fig. 5A). We also studied the expression of Paxillin (PXN), which also participates to the cell adhesion complex. The kinase domain deletion induced a decrease of both phosphorylated (pPXN) and total expression of PXN (Fig. 5B). We performed immunofluorescence experiments using antibodies targeting pFAK, FAK and PXN, which are involved in focal adhesion complexes (Fig. 5C). These results confirm our immunoblots and show a decrease of fluorescent puncta localized at the plasma membrane for pFAK and FAK expressions, as well as for PXN expression, strongly suggesting that the kinase deletion abrogated focal adhesion formation in PANC-1 cells. As it has been shown that p21-activated kinase 1 (PAK1) regulates PDAC cell focal adhesion [24], we studied the impact of the deletion of TRPM7 kinase domain on PAK1 and its main activators (Cdc42, Rac and RhoA) (Fig. 5D). We observed a decrease of mRNA expression for PAK1 and RhoA. These results were confirmed at protein levels with a decrease of both phosphorylated and total PAK1 expression (Fig. 5D). Moreover, we also observed a complete disappearance of RAC expression (Fig. 5D). We also showed by co-immunoprecipitation that PAK1 but not RAC may interact with TRPM7 (Fig. 5E). Using ACC-047 antibody from Alomone Labs which is recommended for IP assays and which recognizes its epitope near the pore domain (on aa residues 1146–1166), we detect two immunostainings corresponding to the full-length protein and possibly to the cleaved TRPM7. Interestingly, we observe an increase of cleaved TRPM7 expression associated to a decrease of full-length TRPM7 expression in ΔK cells. By using Proximity Ligation Assays (PLA), we detected a close protein–protein interaction between TRPM7 and PAK1 in PDAC cells (Fig. 5F). Using the proteinprompt webserver [25], we queried the interactome of the amino acid sequence corresponding to the TRPM7 kinase domain (from AA1098 to 1865, Uniprot Q96QT4). We predicted a physical interaction between TRPM7 kinase domain and PAK1 with 0.71 score corresponding to a good specificity with low false positive rate (*data not shown*). Finally, a reduced number of PLA puncta per cell was detected followed treatment with TG100-115 suggesting that TRPM7 kinase activity is required for interaction between TRPM7 and PAK1 in PDAC cells.

### TRPM7 kinase deletion prevented pancreatic tumor development in vivo

**Fig. 6:**
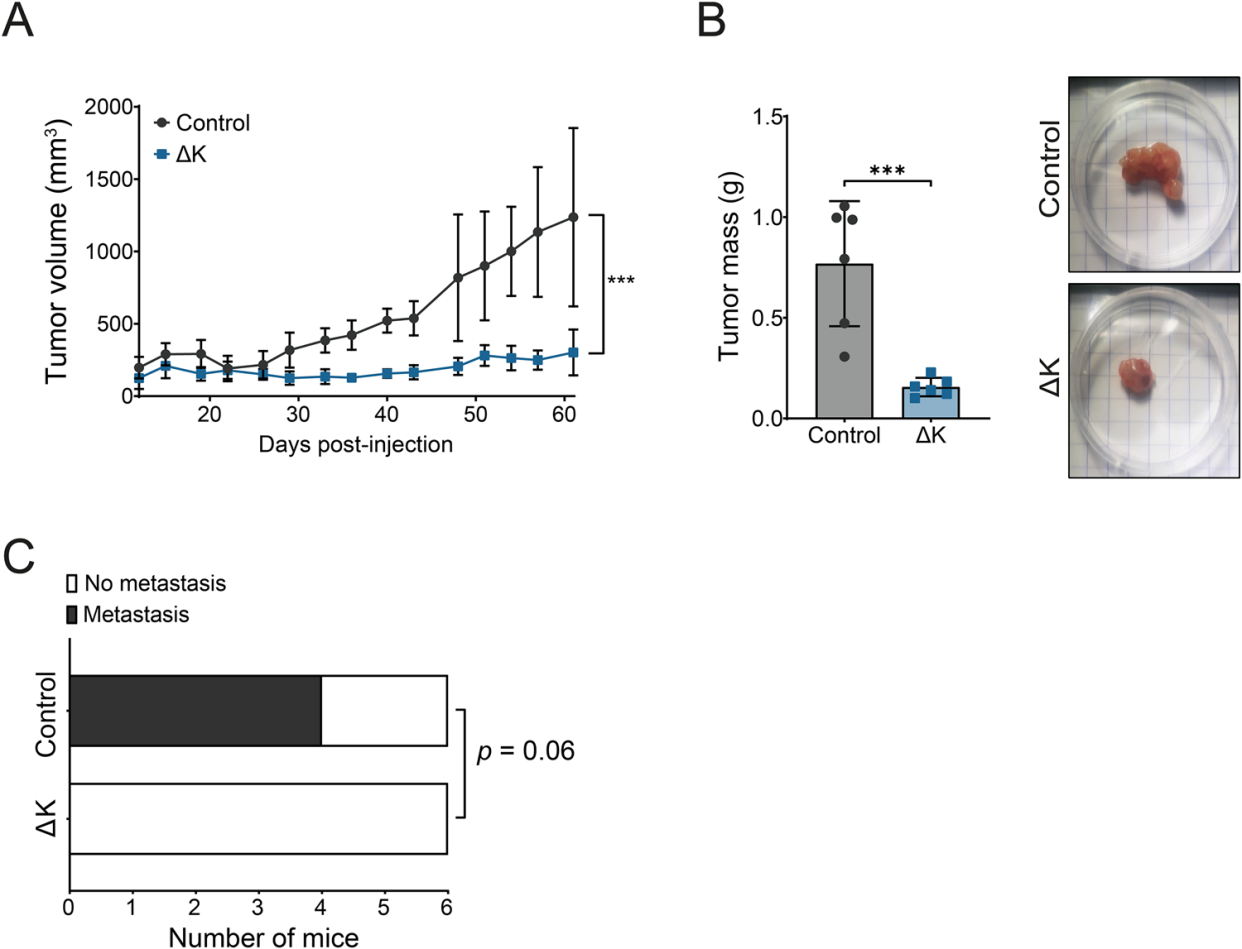
TRPM7 kinase domain deletion prevents pancreatic tumor development in vivo. **A** Monitoring of tumor volume on SCID mice injected with PANC-1 Control (*n* = 6) and ΔK (*n* = 6, *p* < 0.001, Two-way ANOVA) for 60 days. **B** Masses in grams of tumors weighted after mice sacrifice (*n* = 6, *p* < 0.001, Student t-test). **C** Detection of micrometastasis in the liver and lungs of mice injected with PANC-1 Control and ΔK cells (Fisher test). Human GAPDH was used to detect micrometastasis in those organs. Results are shown as mean ± SD. ***p < 0.001.

The role of TRPM7 kinase domain was tested in vivo in a model of xenograft in mice. Tumor growth was monitored for 60 days post injection and we observed a constant increase of tumor volume in control mice from 30 to 60 days but not for mice xenografted with ΔK cells (Fig. 6A). Moreover, the tumor mass after sacrifice was significantly lower in mice xenografted with ΔK cells compared to the control ones (Fig. 6B). The liver and lungs were extracted and human GAPDH mRNA was detected by RT-qPCR in order to highlight the presence of micrometastasis in these organs. Human GAPDH mRNA was detected in 2 livers and 3 lungs of 4 control xenografted mice but, not in mice xenografted with ΔK cells strongly suggesting that PANC-1 cells were unable to disseminate in these organs in the absence of the TRPM7 kinase domain (*p* = 0.06; Fig. 6C). Taken together, our results showed that the TRPM7 kinase domain is required for tumor growth and pancreatic cancer cell dissemination.

### Role of TRPM7 kinase in the MIA PaCa-2 KRAS G12C PDAC cell line

**Fig. 7:**
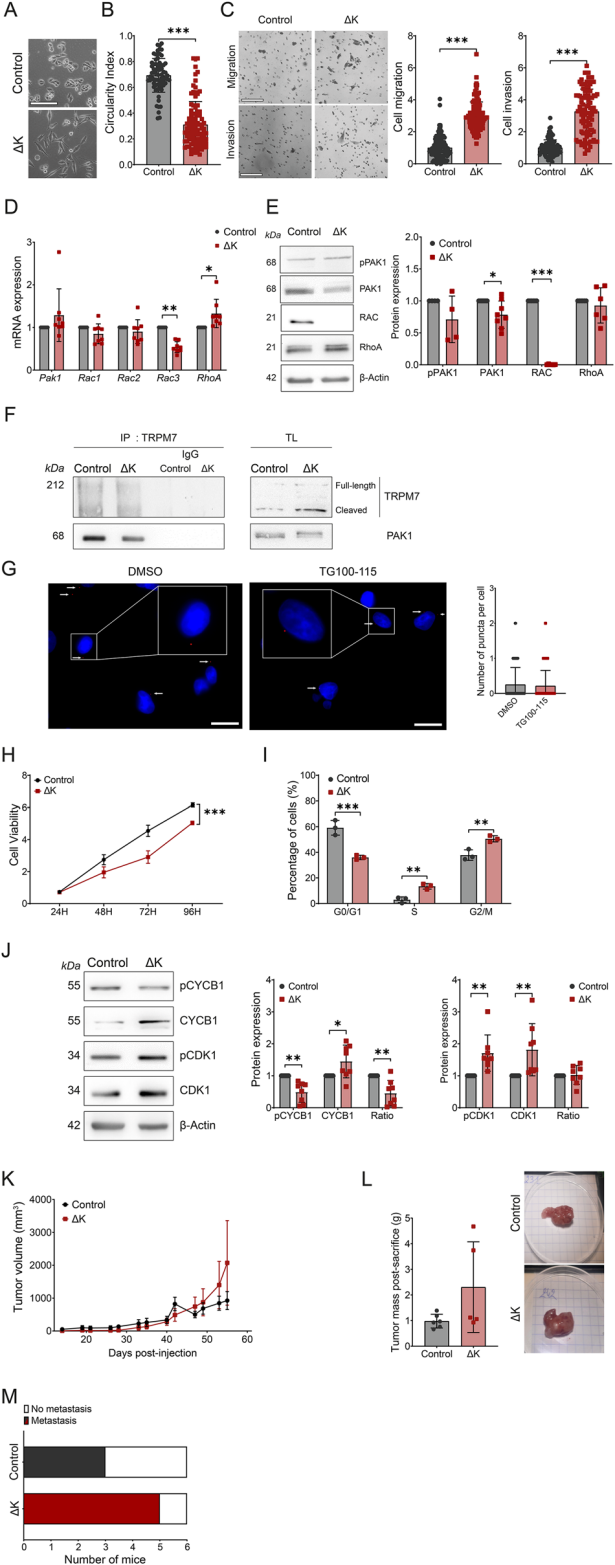
Role of TRPM7 kinase domain in KrasG12C PDAC cell line. **A** Representative morphology of MIA PaCa-2 Control and ΔK cells at ×100 magnification. Scale bar=200 µM. **B** Circularity index calculated for MIA PaCa-2 Control (*n* = 71) and ΔK (*n* = 98) cells (*p* < 0.001, Mann–Whitney test). **C** Cell migration and invasion of MIA PaCa-2 Control and ΔK evaluated in Boyden chambers (*n* = 3, *p* < 0.001, Mann–Whitney test). Scale bar=200 µM. **D** mRNA expression of the RhoGTPase family *Pak1*, *Rac1*, *Rac2*, *Rac3*, *RhoA* in MIA PaCa-2 Control and ΔK cells (*n* = 8, *p* < 0.05 and *p* < 0.01; Two-way ANOVA followed by Šidák’s *post-hoc* test). **E** Protein expression levels of pPAK1 (*n* = 4), PAK1 (*n* = 8*, p* < 0.05, Mann–Whitney test), RAC (*n* = 6, *p* < 0.01, Mann–Whitney test) and RhoA (*n* = 6, Mann–Whitney test in MIA PaCa-2 Control and ΔK cells. **F** Co-immunoprecipitation of TRPM7 with PAK1 in MIA PaCa-2 Control and ΔK cells and their respective positive and negative controls. **G** Proximity Ligation assay of TRPM7 with PAK1 in MIA PaCa-2 cells incubated with DMSO (*n* = 272) or 50 µM TG100-115 (*n* = 277) for 24 h (Mann–Whitney test). **H** Cell viability of MIA PaCa-2 Control and ΔK cells assessed by MTT assay during 96 h (*n* = *3*, *p* < 0.001, Two-way ANOVA followed by Šidák’s *post-hoc* test). **I** Cycle cell distribution of MIA PaCa-2 Control and ΔK evaluated by flow cytometry (*n* = 3, *p* < 0.01 and *p* < 0.001, Two-way ANOVA followed by Šidák’s *post-hoc* test). **J** Expression and phosphorylation levels of CYCB1 and CDK1 proteins in MIA PaCa-2 Control and ΔK cells (*n* = 8, *p* < 0.05 and *p* < 0.01, Two-way ANOVA followed by Šidák’s *post-hoc* test). **K** Monitoring of tumor volume on SCID mice injected with MIA PaCa-2 Control (*n* = 6) and ΔK (*n* = 6) for 54 days (Two-way ANOVA). **L** Masses in grams of tumors weighted after mice sacrifice (*n* = 6, Student *t*-test). **M** Detection of micrometastasis in the liver and lungs of mice injected with MIA PaCa-2 Control and ΔK cells (Fisher test). Human GAPDH was used to detect micrometastasis in those organs. Results are shown as mean ± SD. *p < 0.05; **p < 0.01; ***p < 0.001.

We tested the effect of TRPM7 kinase domain deletion in the MIA PaCa-2 cells that have the rare G12C KRAS mutation present in almost 1–2% of PDAC [21]. In contrast to what we observed for PANC-1, the ΔK cells were less rounded (Fig. 7A, B) and the kinase domain deletion increased the cell migration and invasion in MIA PaCa-2 cells (Fig. 7C). We also observed a decrease of RAC3 mRNA expression (Fig. 7D) and a decrease of both PAK and RAC protein expressions in ΔK cells without modification of RhoA expression (Fig. 7E). PAK1 coimmunoprecipitates with TRPM7 but we do not observe any protein–protein interaction by using PLA (Fig. 7F, G). These results suggest that TRPM7 and PAK1 are in the same protein complex but they do not interact in MIA PaCa-2 cells.

Moreover, the cell viability was reduced in ΔK cells (Fig. 7H). The reduced cell viability observed in ΔK cells was accompanied by a blockade of cell cycle in S and G2/M phases (Fig. 7I). We assessed the expression of cell cycle regulators and we showed a decrease of phosphorylated cyclin B1 (pCYCB1) and an increase of total cyclin B1 (CYCB1) expressions associated with an increase of both phosphorylated and total CDK1 expressions (Fig. 7J). Finally, the tumor growth and the detection of micrometastasis were not affected by the TRPM7 kinase deletion in mice xenografted with MIA PaCa-2 cells (Fig. 7K–M). These results led us to hypothesize that the TRPM7 kinase domain role is cell specific and may depend on the KRAS oncogene mutation status.

## Discussion

We previously demonstrated that TRPM7 is overexpressed in PDAC [9] and regulates cell migration and invasion [8]. Here we show a role for the TRPM7 kinase domain in pancreatic carcinogenesis since (i) TRPM7 kinase domain is required to maintain a mesenchymal phenotype in PDAC cells, (ii) TRPM7 and PAK1 interact in the same protein complexes in PDAC cells, (iii) TRPM7 kinase domain is required for carcinogenesis and cancer cell dissemination in vivo, and iv) the role of TRPM7 kinase is cell specific and may depend on the KRAS oncogene mutation status.

In our study, we used a model of endogenous kinase domain deletion by CRISPR-Cas9 strategy. The kinase domain deletion has been validated by RT-qPCR and by immunoblotting. The deletion was not complete as we detected a TRPM7 kinase domain expression decrease of almost 55% in PANC-1 and almost 70% in MIA PaCa-2 cells. The role of TRPM7 kinase domain on the ion channel function is still under debate. In our model, the kinase domain deletion has no effect on whole-cell MIC currents. These results are in good agreement with recent publication using similar model of TRPM7 kinase deletion in leukemia [26]. Moreover, we have not detected any changes in the TRPM7 current sensitivity to intracellular free Mg^2+^. On the other hand, the TRPM7-kinase dead cells lose their sensitivity to MgATP. Demeuse et al. proposed a hypothetical model of TRPM7 regulation by Mg^2+^ and MgNTP comprising two Mg-interacting binding sites, one for free-Mg^2+^ near the pore and one for MgNTP located in the kinase domain [27]. Based on this hypothetical model, the loss of MgATP sensitivity observed in the ΔK cells may be explained by the absence of the MgNTP binding site. However, Demeuse *et al*. reported that TRPM7 kinase-death cells were more sensitive to both free Mg^2+^ and MgATP due a better exposition of the free-Mg^2+^ binding site [27]. This discrepancy may be explained by the difference between our cell models (HEK293 *vs* PDAC cell lines) and between the strategy used to delete the TRPM7 kinase domain.

As EMT is the first step of the metastasis cascade, we assessed EMT through a combination of molecular markers and cellular properties, as recommended by the EMT International Association [5]. We observed an increase of E-cadherin expression associated with a decrease of vimentin expression in cells lacking the kinase domain. Moreover, some of the core EMT-associated transcription factors (Zeb1, Zeb2, Snai1 and Twist1) were downregulated at a transcriptional level in the ΔK cells. These changes were associated with a decrease of both migratory and invasive properties of cancer cells which strongly suggests that the TRPM7 kinase domain is required to maintain a mesenchymal phenotype in PDAC cells. TRPM7 has already be shown to be involved in EMT of breast cancer cells by regulating calcium signaling and SOX4 transcription factor expression [28, 29]. A growing number of research studies suggest that the EMT program is highly plastic and dynamic during PDAC initiation and progression [30]. Here we show that TRPM7 kinase domain could be a promising target to reverse EMT and to prevent cell invasion. As we used a kinase deletion model, it remains to be fully deciphered whether TRPM7 regulates EMT through the domain kinase itself (by a protein–protein interaction mechanism involving the kinase domain) or through its phosphotransferase activity. We used TG100-115 as an inhibitor of TRPM7 kinase activity [23] and we showed that kinase inhibition decreased both features of cells going in EMT such as cell migration and invasion in PDAC cells, without additive effect on the ΔK cells. However, TG100-115 has been shown to also inhibit the TRPM7 ion currents [23] and we cannot fully exclude that TG100-115 reduced PDAC cell motility and invasion through off-target effects on TRPM7 pore domain. Moreover, TG100-115 is also a potent inhibitor of PI3K p110δ which is involved in the migration of several cancer cell types including mainly breast cancer cells [23]. In addition to the fact that PI3K p110δ has not yet been described in PDAC cell migration, the absence of any inhibitory additive effect of TG100-115 on ΔK cell migration and invasion led us to suggest that TRPM7 kinase domain regulates PDAC cell properties mainly through its phosphotransferase activity.

TRPM7 silencing has been shown to inhibit breast cancer cell migration by increasing cell tension and focal adhesions [15]. Here we show that TRPM7 kinase deletion decreased FAK phosphorylation and paxillin expression suggesting rather an inhibition of focal adhesion number associated with the reduced cell migration. It has been shown that cancer cell focal adhesions are regulated by PAK1 promoting PDAC metastasis [24]. PAK1 is overexpressed in PDAC and its high expression is correlated to reduced overall survival [31]. Moreover, PAK1 is located at the nexus of oncogenic KRAS pathways [31, 32]. Interestingly, we showed a decrease of both PAK1 mRNA and protein expressions in ΔK cells compared to the control ones. This was accompanied by a surprising decrease of RAC and RhoA expressions but we did not observe any effect on Cdc42 expression. Liu et al. reported that a loss of TRPM7 in the *Xenopus* embryo increased RAC activity leading to gastrulation defects [33]. On the other hand, TRPM7 knockdown inhibited RAC activity, lamellipodia formation and polarized movement in fibroblasts [34]. These studies strongly suggest that RAC may be affected downstream of TRPM7 and this was confirmed by our study. Recently, it has been shown that RhoA interacts with TRPM7 through its kinase activity in hepatocellular carcinoma cells [35]. We confirmed the interaction between TRPM7 and RhoA by coimmunoprecipitation in PDAC cells (Fig. S1). Here we showed that PAK1 is also recovered from immunoprecipitates with a TRPM7 antibody but not RAC. This interaction was confirmed by PLA and the treatment with TG100-115 abolished the formation of PLA complexes which strongly suggests that TRPM7 kinase activity regulates the interaction between PAK1 and TRPM7. The interactions between PAK1 and the small GTPases involved in cell motility are complex and some studies demonstrated that PAK1 could act upstream and downstream of RAC activation [36]. It is tempting to speculate that TRPM7 kinase activates PAK1 leading to RAC activation, cell focal adhesion assembly and cell migration. Importantly, we confirmed the importance of TRPM7 kinase domain in PDAC carcinogenesis in vivo in a model of mouse xenograft. Tumor growth and micrometastasis detection were lower in animals injected with ΔK cells compared to the controls. Based on these results, TRPM7 kinase inhibition could be proposed as a promising strategy to treat PDAC progression and metastasis.

Surprisingly, we observed opposite effects when we tested the impact of TRPM7 kinase deletion in MIA PaCa-2 which have the KRAS G12C mutation (1–2% of all PDAC). In these cells, the TRPM7 kinase deletion induced a cell elongation associated with enhanced cell migration and invasion. A similar phenotype was obtained following treatment of control cells with TG100-115 indicating that the TRPM7 kinase prevents the acquisition of invasive phenotype in MIA PaCa-2 cells. We previously showed that TRPM7 silencing inhibited MIA PaCa-2 cell migration and invasion through [Mg^2+^]_i_ reduction [8]. Interestingly, TRPM7 silencing fully abolished the enhanced cell migration and invasion in the MIA PaCa-2 ΔK cells (Fig. S2). This led us to hypothesize that TRPM7 channel and kinase functions regulate the MIA PaCa-2 cell motility through opposite mechanisms. These opposite effects of TRPM7 kinase on PDAC cell migration highlight the importance of taking cellular heterogeneity into account when studying PDAC. Thus, PDAC cell lines can have very different phenotypes, and the MIA PaCa-2 cells have lower migratory and invasive capacities than PANC-1, as previously reported [37, 38]. The EMT has been also assessed in MIA PaCa-2 cells (Fig. S3). We observe that kinase deletion induced an elongated morphology and the cells were less clustered (A and B panels). Both *Cdh1* and *vimentin* mRNA expression were reduced in ΔK cells (C panel) but this was not confirmed at protein level (D panel). Regarding the EMT core transcription factors, only *Zeb1* and *Twist1* expression were increased in ΔK cells (E panel). As already shown in Fig. 7 of the manuscript, kinase deletion stimulated both MIA PaCa-2 cell migration and invasion (Fig. 7C). Taken together, these data suggest that TRPM7 kinase partially regulates EMT in MIA PaCa-2, and confirmed that TRPM7 kinase is involved in EMT process. Similarly, to the PANC-1 ΔK cells, we observe a decrease of both PAK1 and RAC expressions in the MIA PaCa-2 ΔK cells. However, the phosphorylated PAK1 expression is maintained, albeit at a low level in MIA PaCa-2 ΔK cells. We also detected an interaction between PAK1 and TRPM7 by coimmunoprecipitation but this was not confirmed by PLA assays. Interestingly, similar results were found in non-cancer HPNE cell line (Fig. S4). This suggests that PAK1 and TRPM7 are probably in a common multimolecular complex but they do interact more closely only in PANC-1 cells. We hypothesize that TRPM7 may regulate PDAC cell migration depending of the KRAS mutation. To test this hypothesis, we treated with TG100-115 the AsPC-1 PDAC cells that have the G12D KRAS mutation, and we observed a decrease of both cell migration and invasion as for the PANC-1 cells (Fig. S5).

On the other hand, the TRPM7 kinase deletion decreased the MIA PaCa-2 cell proliferation by accumulating the cells in G2/M phase of the cell cycle and by inhibiting the cyclin B1 activation. The opposite effects of TRPM7 kinase deletion on cell proliferation and migration depending of cell lines may be explained at least partially by the modulation of different signaling pathways. Clearly, it appears that the Rho/Rac ratio is highly different in response to the deletion of the kinase domain in both cell lines. Moreover, we showed for example that the ERK signaling pathway is activated in the MIA PaCa-2 ΔK cells but not in the PANC-1 ΔK cells (Fig. S6A). Interestingly, Koujima *et al*., highlighted the importance of ERK signaling pathway in PDAC cell invasiveness and metastasis [39]. On the other hand, the EGFR expression is almost fully inhibited in the MIA PaCa-2 ΔK cells (Fig. S6B) which could explain the effect of the kinase deletion on cell proliferation. We did not observe any effect on the AKT expression in our models (Fig. S6C), although it has been shown that TRPM7 kinase regulates AKT signaling in chronic myeloid leukemia cells [26]. Despite these effects at cellular and molecular levels, we did not observe any effect of the TRPM7 kinase deletion on tumor growth nor on micrometastasis detection in mice xenografted with MIA PaCa-2 cells. This suggests that TRPM7 kinase deletion has no impact on KRAS G12C tumor progression nor metastasis dissemination. Some KRAS G12C inhibitors such as sotorasib and adagrasib have been developed and are approved for clinical use [40]. The effect of TRPM7 kinase deletion in combination with KRAS K12C inhibitors need to be tested as a possible approach to target tumors with G12C KRAS mutation.

**Fig. 8:**
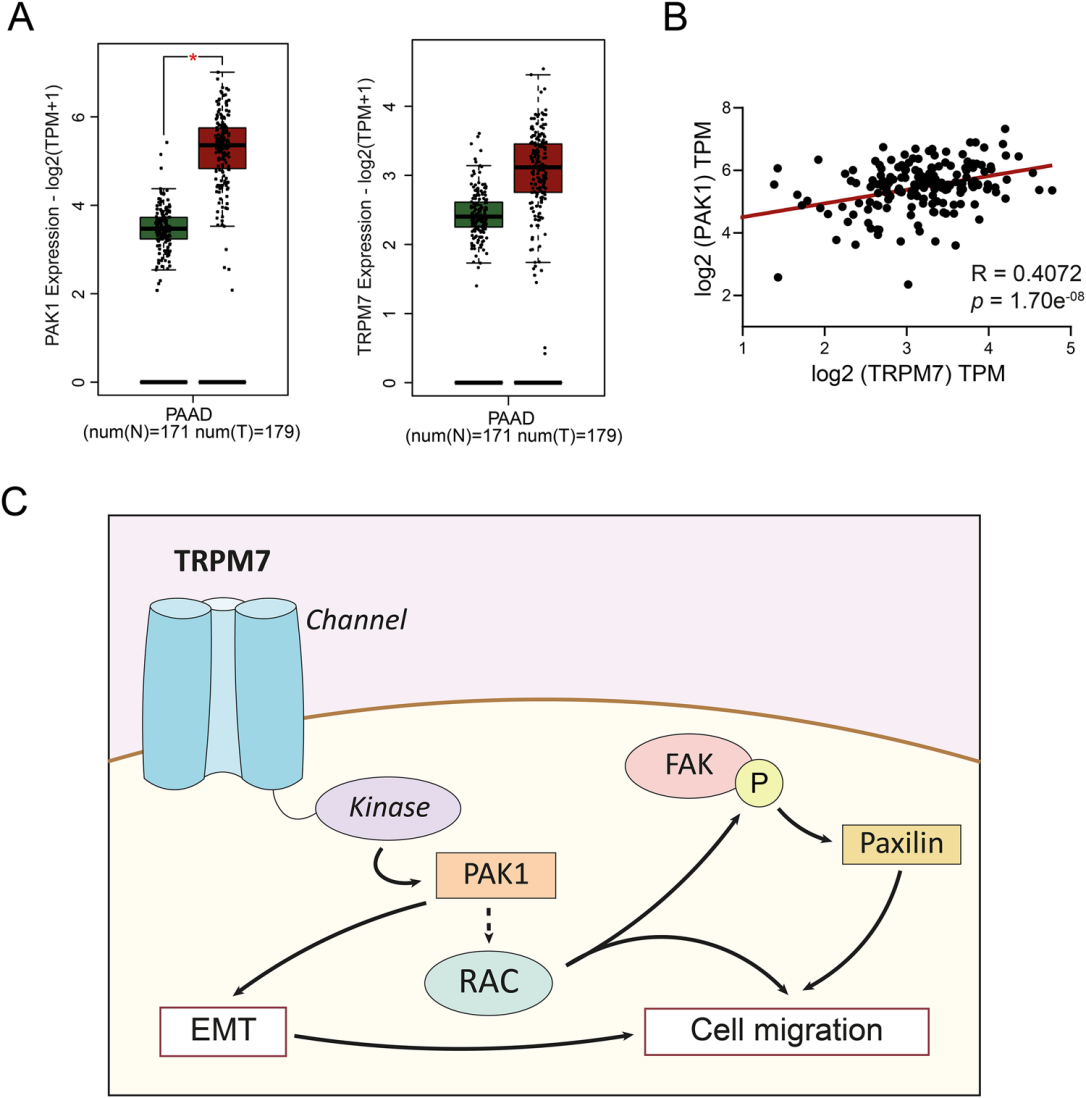
Analysis of TRPM7 and PAK1 expression in TCGA and graphical abstract. **A** Relative mRNA expression of PAK1 and TRPM7 in healthy patients (*n* = 171) and PDAC patients (*n* = 179) using PAAD cohort in GEPIA2 and GTEx datasets (*p < 0.01). **B** Pearson correlation R values were calculated for PAK1 and TRPM7 in PAAD dataset using cBioPortal website. **C** Summary of the major findings presented in this article for PANC-1 cell line.

In a clinical point of view, we confirmed that PAK1 is overexpressed in PDAC tissues by analyzing the TCGA-PAAD/GTEX GEPIA transcriptomic database while TRPM7 transcriptomic expression was not significantly different between cancer and non-cancer tissues (Fig. 8A). Interestingly, a positive correlation was found between the expression of PAK-1 and TRPM7 in PDAC tissues (Fig. 8B). The presence of TRPM7-PAK1 complexes in PDAC tissue and its clinical significance needs further investigations. In conclusion, we showed that the TRPM7 kinase domain interacts with PAK1 and regulates FAK phosphorylation, paxillin expression, and cell migration in PDAC (Fig. 8C).

## Materials and methods

All the references and suppliers’ products, reagents, software and machines were listed in supplementary data (Table 1).

### Cell lines

Human pancreatic cancer cell lines PANC-1 (ATTC, #CRL-1469) and MIA PaCa-2 (ATCC, #CRL-1420) cell lines were used in this study. Cells were cultured in Dulbecco’s modified Eagle’s medium with a 10% (v/v) FBS supplementation. PANC-1 and MIA PaCa-2 were grown at 37 °C in a humidified atmosphere with 5% CO_2_ and were trypsinized once per week with trypsin-EDTA.

PANC-1 and MIA PaCa-2 delta-kinase (ΔK) and their corresponding control stable cell lines were established using the CRISPR/Cas9 editing strategy. Briefly, a single guide (sg)RNA was designed using CRISPOR software (http://crispor.gi.ucsc.edu/) to target the TRPM7 kinase domain between exons 33 and 34. The targeting sequence was cloned into a TopoTA vector containing the sgRNA backbone under the U6 promoter (derived from Addgene plasmid #58766 deposited by Dr T. Yamamoto). A homology-directed repair donor plasmid containing the puromycin resistance gene and a transcriptional terminator (kind gift of Dr. M. Wassef, Paris) [41] was constructed with homology arms to TRPM7. This vector was co-transfected with pCas9-GFP (Addgene plasmid #44719 deposited by Dr K. Musunuru) and either the cloned sgRNA TRPM7 or, as control, the sgRNA AAVS1-Target 2 [42] using Lipofectamine 3000 Transfection reagent according to the manufacturer’s instructions. Positive PANC-1 and MIA PaCa-2 clones were then selected with a chronic puromycin treatment of 5 µg/mL and 2 µg/mL, respectively. Stable deletion of the kinase domain was quantified by RT-qPCR and confirmed by western-Blot using TRPM7 antibody raised against its kinase domain in both cell lines.

### RT-qPCR

PANC-1 and MIA PaCa-2 total RNA was extracted using TRIzol reagent protocol. RNA concentration and purity were assessed using NanoDrop spectrophotometer and 2 µg of total RNA was then reverse transcribed into cDNA using the High-Capacity cDNA RT-kit following the manufacturer’s instructions. Quantitative real-time PCR (RT-qPCR) was performed using PowerUp SYBR Green Master Mix on QuantStudio™ 7. All the sequences of the primers used are listed in Table S3 (Supplementary methods). Relative gene expression was calculated Pfaffl method [43]. POP4 and MRPL19 were used as housekeeping genes for the two cell lines using Genorm method [44].

### Western blotting

PANC-1 and MIA PaCa-2 proteins were extracted using RIPA buffer supplemented with protease inhibitors. Lysates were incubated on ice and centrifuged at 12,000 × *g* for 15 min at 4 °C and protein concentration was determined using the DC Protein Assay following manufacturer’s instructions. Equal amounts of proteins were separated on SDS-PAGE and transferred to a nitrocellulose membrane. Membranes were blocked in 5% (w/v) non-fat milk for 2 h at room temperature and incubated with primary antibodies (listed in Table 2, Supplementary methods) at 4 °C overnight. The next day, membranes were incubated for 2 h at room temperature with corresponding HRP-conjugated secondary antibodies. Protein bands were visualized using enhanced chemiluminescence (ECL) detection system on XRS Chemidoc Imager and quantification was performed using Quantity One software. β-Actin and GAPDH were used as loading controls. For western blots, we used the ab245408 antibody from Abcam, which recognizes its epitope on aa residue 1800 in the kinase domain at the C-terminus. In consequence, immunoblotting only detect the full-length TRPM7 expression and not the cleaved protein. For the co-immunoprecipitation experiments, we used the ACC-047 antibody from Alomone Labs which is recommended for IP assays and which recognizes its epitope near the pore domain (on aa residues 1146–1166), detecting both full-length and cleaved TRPM7.

### Electrophysiology

TRPM7 activity in PANC-1 and MIA PaCa-2 cells was evaluated by whole-cell patch-clamp recording. Cells were placed in a bath solution composed of 150 mM Na-Gluconate, 5 mM K-gluconate, 2 mM Mg-gluconate, 2 Ca-gluconate, 10 mM HEPES, 5 mM Glucose, 5 mM TEA, pH 7.4 at room temperature. Patch pipettes with a resistance ranging from 3 to 6 mΩ were filled with an intrapipette solution composed of 8 mM Na-gluconate, 10 mM HEPES, 10 mM EGTA, 145 mM Cs-Gluconate, pH 7.2. A gigaseal was formed between the pipette and the cell membrane to break into the whole-cell configuration. For Mg^2+^ and ATP experiments, free [Mg^2+^] was calculated using MAXCHELATOR software (https://somapp.ucdmc.ucdavis.edu/pharmacology/bers/maxchelator.htm). Membrane potential was held at −40 mV and a protocol of ramp depolarization from −100 mV to +100 mV for 350 ms every 10 s. Signals were then filtered at 1 kHz and digitized at 5 kHz using an Axopatch 200B amplifier and analyzed with pClamp 10 and Clampfit. MIC currents were recorded after intracellular media dialysis by the intrapipette solution and expressed as current densities in pA.pF^−1^.

### Cell migration and invasion assays

Cell migration was performed using 8 µm pore size Boyden chambers and invasion with Boyden chambers coated with Matrigel. 4 × 10^4^ PANC-1 or MIA PaCa-2 cells were seeded in the upper compartment in growth medium with 0.1% FCS and the lower compartment was filled with growth medium supplemented with 10% FCS. After 24-h of incubation at 37 °C, the remaining cells in the upper compartment were removed by scrubbing. Migrating or invading cells were washed in PBS, fixed with methanol, and stained with haematoxylin solution. Cell migration/invasion was then quantified by counting 20 different fields at x400 magnification under an inverted microscope. An MTT test was carried out at each experiment to avoid seeding or counting errors.

### Co-Immunoprecipitation (Co-IP)

Co-immunoprecipitation was performed to assess protein–protein interactions using Bio-Rads’ SureBeads Magnetic Beads A following the manufacturer’s instructions. Magnetic protein A beads were washed and the primary antibody (TRPM7, 1 µL) was incubated with the beads for 30 min. Negative controls were realized by using anti-Rabbit IgG. Beads were then washed and 500 µg protein lysate was added at room temperature for 2 h. After PBS washes, beads were eluted by adding Laemlli buffer and incubating them for 10 min at 70 °C. Eluates and their corresponding total lysates were analyzed by Western-blot to detect the presence of co-immunoprecipitated proteins.

### Proximity ligation assay (PLA)

Proximity ligation assay was performed to assess protein–protein interactions using the Duolink In Situ kit according to the manufacturer’s instructions. Briefly, PANC-1 and MIA PaCa-2 were seeded onto Labtek® chambers and allowed to adhere overnight. After treatment, cells were fixed and permeabilized with frozen methanol. After blocking with blocking buffer for 1 h at 37 °C, cells were incubated with primary antibodies (TRPM7, 1/500^e^; PAK1, 1/500^e^) for 2 h at 37 °C. Cells were then washed and incubated with the respective PLA probes for 1 h at 37 °C. Ligation was conducted for 30 min and amplification for 100 min at 37 °C. Cell nuclei were stained with DAPI and fluorescent PLA signals were imaged using a fluorescence microscope. Image analysis and quantification of PLA dots per cell were performed using ImageJ software.

### Immunofluorescence

PANC-1 cells were seeded onto Labtek® chambers and allowed to adhere overnight. Cells were fixed and permeabilized with frozen methanol and non-specific sites were blocked with a PBS-BSA 3% solution. Cells were incubated with primary antibodies (pFAK, tPXN, tFAK, 1/100e) diluted in PBS-BSA 1% overnight at 4 °C, washed with PBS and then incubated with secondary antibodies (1/200e) diluted in PBS-BSA 3% in the dark for 1 h. Cell nuclei were stained with DAPI and fluorescent signals were imaged using a confocal microscope (Stellaris 5, Leica, Nanterre, France). Image analysis was performed using ImageJ software.

### Mice xenografts and micrometastasis detection

Xenograft models were established to study tumor growth and progression using PANC-1 and MIA PaCa-2 cells. Eight-week-old male severe combined immunodeficient (SCID) mice were randomly assigned in two groups and subcutaneously injected with 1 × 10^6^ cells resuspended in a 1:1 mixture of serum-free medium and Matrigel. Tumor volume and mice weight were then followed 14 days after injection, twice a week. Blind measurements of tumor volumes were realized during the experiment. Tumor size was evaluated weekly by measuring the length (L) and the width (W) and tumor volume was calculated with the formula (W2 × L/2). 60 days post injection, mice were sacrificed by cervical dislocation, and tumors, lungs, and liver were excised for molecular analysis. RNA extraction was realized on mice lungs and liver using NucleoSpin RNA Midi kit following the manufacturer’s instruction and RNA was then reverse transcribed into cDNA. To detect micrometastasis, we used human GAPDH (hGAPDH) and mouse GAPDH (mGAPDH) primers (sequences listed in Table 3, Supplementary methods).

### Ethics approval

All animal experiments were conducted following ethical guidelines and approved animal care protocol (Apafis #20220331141694) by the ethics committee CEEA75 (“Comité d’Ethique en Expérimentation Animale”).

### Analysis of PDAC datasets

PAK1 and TRPM7 expression was extracted from GEPIA2 [45]. GEPIA2 uses expression data from the Cancer Genome Atlas (TCGA) and from the Genome Tissue Expression (GTEx), allowing to compare PAK1 and TRPM7 expression in PDAC patients (PAAD cohort) and healthy patients. The platform cBioPortal [46–48] was used to retrieve mRNA expression from PAAD cohort to calculate Pearson correlation coefficient (R value) and *p-*values for PAK1 and TRPM7.

### Statistical analysis

Statistical analyses and figures were performed using GraphPad Prism version 9. All data are presented as mean ± standard deviation (SD) from at least three independent experiments (*n* ≥ 3). For experiments like patch-clamp, calcium imaging, circularity index study and proximity ligation assays, n will be representing the number of cells. Xenograft cohort sizes were calculated by EpiR package 0.9-30 using biostatgv tool (alpha risk = 0.05). No animal was excluded from the analysis. For comparisons between two groups, the unpaired Student t-test was used for normally distributed data, while the Mann–Whitney test was applied for non-normally distributed data. For comparison between more than two groups, one-way analysis of variance (ANOVA) was used for normally distributed data and Kruskal–Wallis test for the non-normally distributed data. For experiments with more than one parameter studied, two-way ANOVA was performed. Šidák’s test was used as a *post-hoc* test to determine statistical significance between groups. A *p*-value < 0.05 was considered statistically significant.

## Supplementary information

Supplemental Data

Original western blots

## Data Availability

The datasets generated and/or analyzed during the present study are available from the corresponding author upon reasonable request.
