# Supplementary material for: The kinase domain of TRPM7 interacts with PAK1 and regulates pancreatic cancer cell epithelial-to-mesenchymal transition
Source: Cell Death Dis. 2025 Apr 24;16(1):335. doi: 10.1038/s41419-025-07665-2 (PMC12022261; doi:10.1038/s41419-025-07665-2)
Supplement: Supplementary file 1 — Supplemental Data [file 41419_2025_7665_MOESM1_ESM.pdf]

Supplemental Data:

Supplemental Material and Methods:

Manganese quenching:

Calcium influx was assessed using manganese quenching assay. 70 000 PANC-1 or 40 000 MIA PaCa-2 cells were seeded and incubated for 48 hours. Cells were then incubated with 3 µM Fura-2 AM for 45 minutes at 37°C. After loading, fluorescence was measured at the isosbestic point (360 nm excitation) with recordings taken every second for a total duration of 3 minutes. During this protocol, cells were washed with 2 mM Ca^{2+} solution for 1 minute and then perfused with 2 mM Mn^{2+} solution. The quenching was evaluated by calculating the slope, relative to the initial baseline fluorescence.

MTT viability assay:

Cell viability was assessed using MTT assay. $4x10^4$ PANC-1 and MIA PaCa-2 cells were seeded and allowed to adhere for 24 hours. 800 µL of MTT solution (5 mg/mL) was added and incubated for 1 hour at 37°C. The medium was then removed and formazan crystals formed were dissolved in 800 µL of dimethyl sulfoxide (DMSO). The optical density was measured at 570 nm using TECAN spectrophotometer.

Wound healing:

To assess collective cell migration, wound healing assay was performed. $6x10^6$ PANC-1 or MIA PaCa-2 cells were seeded in 6-well plates and allowed to adhere and reach confluence. A sterile 200 µL pipette tip was used to create a wound in the cell monolayer. Cells were washed with PBS to remove all the detached cell and then incubated with medium. The wound was photographed immediately after the experiment (T – 0H) and after 24 hours (T – 24H) using an inverted microscope. Wound areas were quantified using ImageJ software, and the percentage of wound closure was calculated using the following formula:

% of Wound Closure = (Area0H – Area24H) / Area0H x 100

Cell cytometry:

Flow cytometry was performed to evaluate cell cycle distribution. 300 000 MIA PaCa-2 or PANC-1 cells were seeded and incubated for at least 24 hours. Cells were harvested with trypsin-EDTA, washed twice with PBS, once with PBS-EDTA (5 mM) and then fixed with 70% ethanol. After fixation, cells were washed with PBS and resuspended with RNase A and propidium iodide for 30 minutes in the dark. Flow cytometry was performed with Cytometer Accuri C6 and the percentage of cells in the G1, S and G2/M cycle was estimated using Cyflogic software.

Table S1: List of chemicals, products, software and machines used for the study.

Product name	Supplier	Reference
Cell culture		
DMEM High Glucose	Gibco	31966021

Puromycin	Gibco	A11138
Fœtal Bovine Serum	Gibco	10270<br>Lot 42Q9079K
Lipofectamine™ 3000	FisherScientific	15232475
Invitrogen™ TOPO™ TA Cloning™ Kit	FisherScientific	10587163
Trypsine EDTA	Gibco	25300-054
TG100-115	Selleck Chem	S1352
Products used in patch-clamp and Mn^{2+} quenching assay		
Magnesium chlorure	Sigma	M2670
Potassium chlorure	Sigma	P3911
Sodium chlorure	Sigma	S3014
Fura-2	Sigma	F0888
Calcium gluconate	Sigma	C8231
Magnesium gluconate	Sigma	344435
Potassium gluconate	Sigma	G500
Sodium gluconate	Sigma	S2054
Glucose	Sigma	G5767
HEPES	Sigma	H3375
TEA	Sigma	T2265
Hematocrit glass	Hirschmann®	9100160
Migration and invasion		
Hematoxylin	Sigma	1.09253
Boydens chambers - Invasion	FisherScientific	11553570
Boydens chambers - Migration	FisherScientific	10136410
Methanol	Sigma	32213
Cytometry and viability		
DMSO	Sigma	276855
EDTA	Sigma	E5135
Propidium Iodure	FisherScientific	P1304MP
MTT	FisherScientific	F11939
Biochemistry		
Acrylamide/bis acrylamide, 40% solution	Sigma	A7168-100
Ammonium Persulfate (APS)	Fisher	BP179-25
DC Protein Assay Reagent A	BioRad	5000113
DC Protein Assay Reagent B	BioRad	5000114
DC Protein Assay Reagent S	BioRad	5005115
Sodium Deoxycholate	Sigma	D6750
ECL RevelBlOt Intense	Ozyme	OZYB002
Glycerol	Sigma	G5516
Glycin	Sigma	G8898
N,N,N′,N′-Tetramethylethylenediamine (TEMED)	Sigma	T22500
Sodium Orthovanadate - Na_3VO_4	Sigma	S6508-10G
Ponceau Red	Sigma	P3504
Bromophenol Blue	Sigma	B8026
Sodium Dodecyl Sulfate (SDS) - powder	Sigma	L4390
Sodium Dodecyl Sulfate (SDS) - solution	Sigma	71736

TRIZMA Base	Sigma	T1503
Bovine Serum Albumin (BSA)	FisherScientific	BP9701
Molecular weight loading marker	BioRad	1610374
Molecular biology		
TriReagent (Trizol)	Ambion	15596018
2-BromoChloroform	Sigma	B9673
Isopropanol	Sigma	I9516
High-capacity cDNA RT kit	Applied Biosystems	4368814
PowerUp SYBR Green Master Mélange	Applied Biosystems	A25742
RNase away®	Sigma	83931
NucleoSpin RNA Midi kit	MACHEREY-NAGEL	740962.20
Softwares		
Origin 2018	OriginLab Corporation, Northampton, USA	
GraphPad 9.5	GraphPad Software, Boston, USA	
Quantity One	BioRad, Marnes-la-Coquette, France	
ClampFit	Molecular Devices Inc, San José, USA	
ImageJ	https://imagej.nih.gov/ij/index.html	
ZEN 3.5 Blue edition	Carl Zeiss S.A.S., Rueil Malmaison, France	
Cyflogic	CyFlo Ltd, Turku, Finland	
MetaFluor	Molecular Devices Inc, San José, USA	
iControl	Tecan France S.A.S.U., Lyon, France	
NanoDROP	Thermofisher, Life Technologies SAS, Villebon-Sur-Yvette, France	
Design & Analysis Software (DA2)	Thermofisher, Life Technologies SAS, Villebon-Sur-Yvette, France	
Machines and equipment		
AxoPatch 200B	Molecular Devices Inc, San José, USA	
Micropipette Puller P-30	Sutter Instruments, Novato, USA	
Micromanipulator MP230	Sutter Instruments, Novato, USA	
Cytometer Accuri C6	BD Biosciences, Rungis, France	
Spectrophotometer NanoDrop 2000	Thermofisher, Life Technologies SAS, Villebon-Sur-Yvette, France	
Inverted microscope – Fluorescence	Thermofisher, Life Technologies SAS, Villebon-Sur-Yvette, France	
Monochromator Polychrome V	FEI, Life Technologies SAS, Villebon-Sur-Yvette, France	
Inverted microscope – Nikon Eclipse TS100	Carl Zeiss S.A.S., Rueil Malmaison, France	
Camera - AxioCam 208 Color, ZEISS	Carl Zeiss S.A.S., Rueil Malmaison, France	
TECAN Spectrophotometer	Tecan France S.A.S.U., Lyon, France	

Western Generators	BioRad, Marnes-la-Coquette, France
XRS Imager Chemidoc	BioRad, Marnes-la-Coquette, France
STELLARIS 5 Confocal Microscope – Immunofluorescence	Leica, Nanterre, France

Table S2: References and dilutions of antibodies used in the study

Protein	kDa	Supplier	Reference	Dilution
TRPM7 (coIP)	212	Alomone Labs	ACC-047	1µg
TRPM7 (Western-Blot)	212	Abcam	ab245408	1/1000
TRPM7 (PLA)	212	Sigma-Merck	SAB5200032-100UG	1/500
Vimentin	58	ThermoFisher	MA5-16409	1/10000
E-cadherin	~ 125	Abcam	ab76055	1/1000
Rac1/2/3	21	Cell Signalling	2465	1/1000
Cdc42	21	Cell Signalling	2466	1/1000
p-PXN (Y118)	68	Cell Signalling	2541S	1/1000
PXN	68	SantaCruz	sc-365379	1/1000
PAK1	68	Cell Signalling	26062	1/1000
FAK	125	Cell Signalling	3585	1/1000
p-FAK (Y397)	125	Cell Signalling	8556	1/1000
RhoA	21	Cell Signalling	2117	1/1000
p-AKT (Ser473)	60	Cell Signalling	9271	1/1000
AKT	60	Cell Signalling	9272	1/1000
p-ERK	42	Cell Signalling	9101	1/1000
ERK	42	Cell Signalling	9102	1/1000
p-EGFR (Tyr1068)	175	Cell Signalling	3777	1/1000
EGFR	175	Cell Signalling	4267	1/1000
GAPDH	37	Cell Signalling	97166	1/2000
β-Actin	44	Cell Signalling	sc-1615	1/20000

Table 3: Primers used in the study

Gene	Sequence F / R (5’ → 3’)
POP4	ACCGCCTGAAAGTTATCCCC
	TTCTTCGCAGACCGTTCACT
MRPL19	TCAGCCAGTTTCTGGGGATT
	GCAAATCTCGACACCTTGTCCT
TRPM7 (Kinase)	TCCATTTACACCTGTGCCTC
	GGGTGAACTCTCTTCCAAACG
TPRM7 (Channel)	CTGTGCCTCCAAGAGGGGAG
	CAGAGGCCTAGTTGTGACCA
Vimentin	AGGAGGAAATGGCTCGTCAC
	AGGCAGAGAAATCCTGCTCTC
(E-cadherin) CDH1	GTTTCTTCGGAGGAGAGCGG
	CTGGCTCAAGTCAAAGTCCTGG
RhoA	CCCCAGAAGTCAAGCATTTC
	TGCCTTCTTCAGGTTTCACC
RAC1	CATTGTTGTGCCGAGAACAC
	GAGTTCAATGGCAACGCTTC

RAC2	CATTGTTGTGCCGAGAACAC
	GAGTTCAATGGCAACGCTTC
RAC3	GAGAATGTTCGTGCCAAGTG
	TCAATGGTGTCCTTGTCGTC
CDC42	CTCAACCCAAAAGGAAGTGC
	GAGGCTTTCAAACAGGATGG
PAK1	ACCCAATAGTGTTCCCATGC
	TTTCCCCGACTTCTCAACAC
ZEB1	GCCAATAAGCAAACGATTCTG
	TTTGGCTGGATCACTTTCAAG
ZEB2	TTTCAGGGAGAATTGCTTGA
	CACATGCATACATGCCACTC
SNAI1	GAATACCTCAGCCTGGGTGC
	CGGACATGGCCTTGTAGCAG
TWIST1	AAACTACAGCGAACTGGACACA
	GCCCCAAAGATGAGGAGTATC
GAPDH (human)	CCACATCGCTCAGACACCAT
	CCAGGCGCCCAATACG
GAPDH (mouse)	AGGTCGGTGTGAACGGATTTG
	TGTAGACCATGTAGTTGAGGTCA

Figure S1:

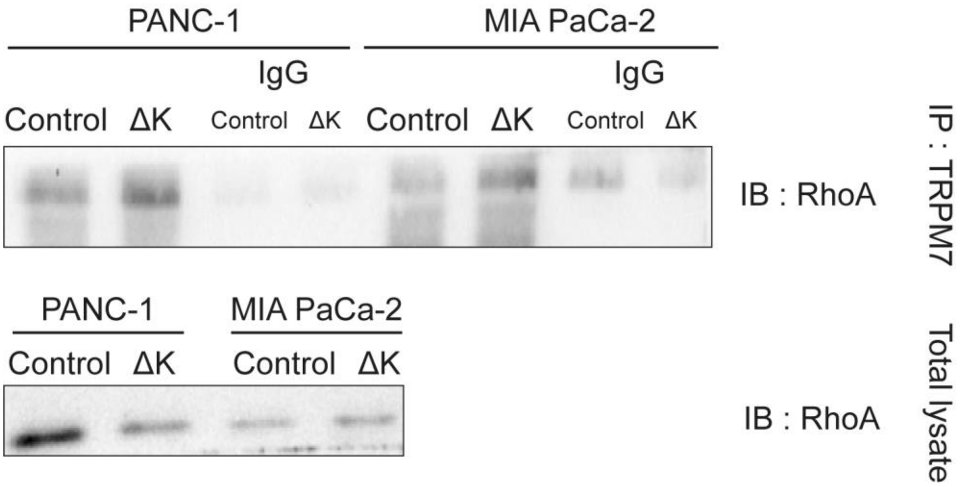

Figure S1: Co-immunoprecipitation of TRPM7 with RhoA in PANC-1 (n=3) and MIA PaCa-2 (n=3) Control and ΔK cells and their respective negative controls.

Figure S2:

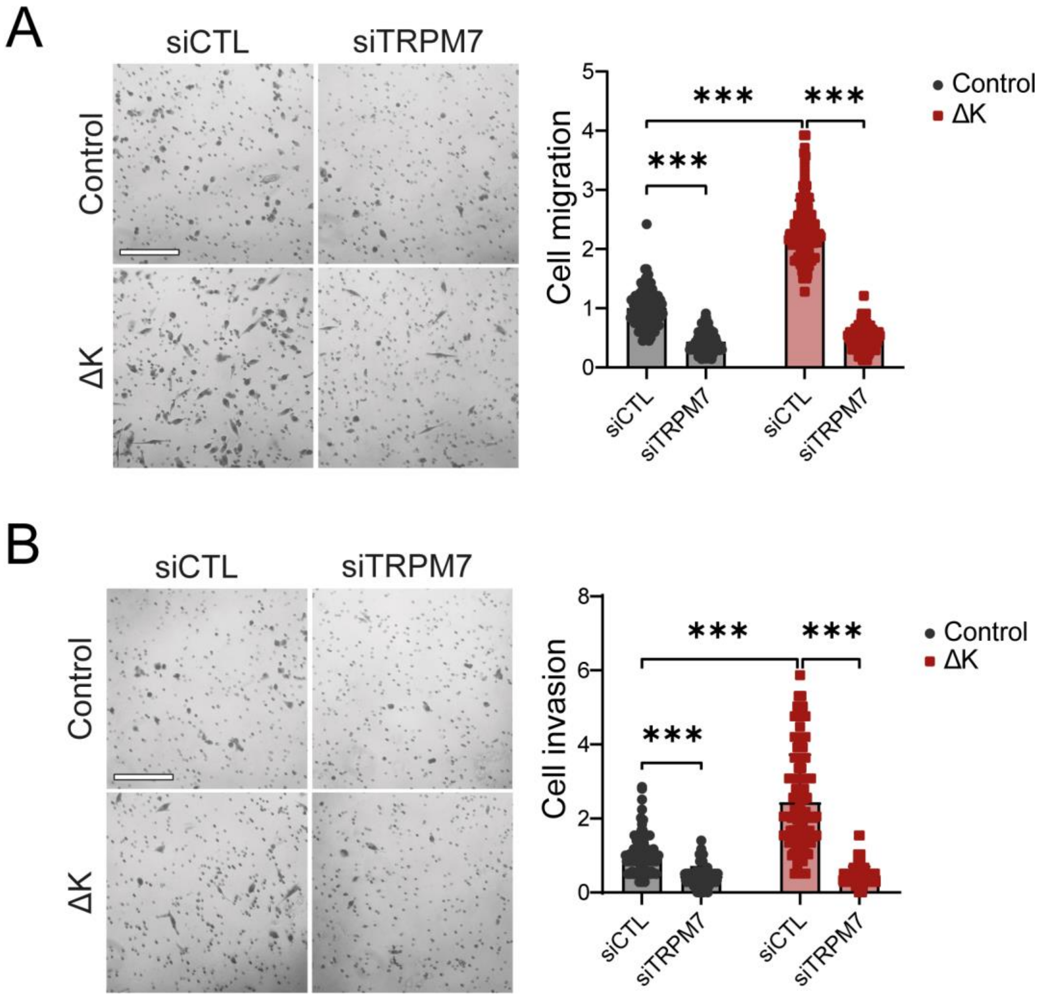

Figure S2: Effect of TRPM7 silencing on MIA PaCa-2 cell migration and invasion. **A** Cell migration of MIA PaCa-2 Control and ΔK siCTL and siTRPM7 evaluated in Boyden chambers (n=3, $p<0.001$, Two-way ANOVA followed by Šidák's *post-hoc* test). **B** Cell invasion of MIA PaCa-2 Control and ΔK siCTL and siTRPM7 evaluated in Boyden chambers coated with Matrigel (n=3, $p<0.001$, Two-way ANOVA followed by Šidák's *post-hoc* test). Scale bars are corresponding to 200 µm. Results are shown as mean±SD. ***$p<0.001$.

Figure S3:

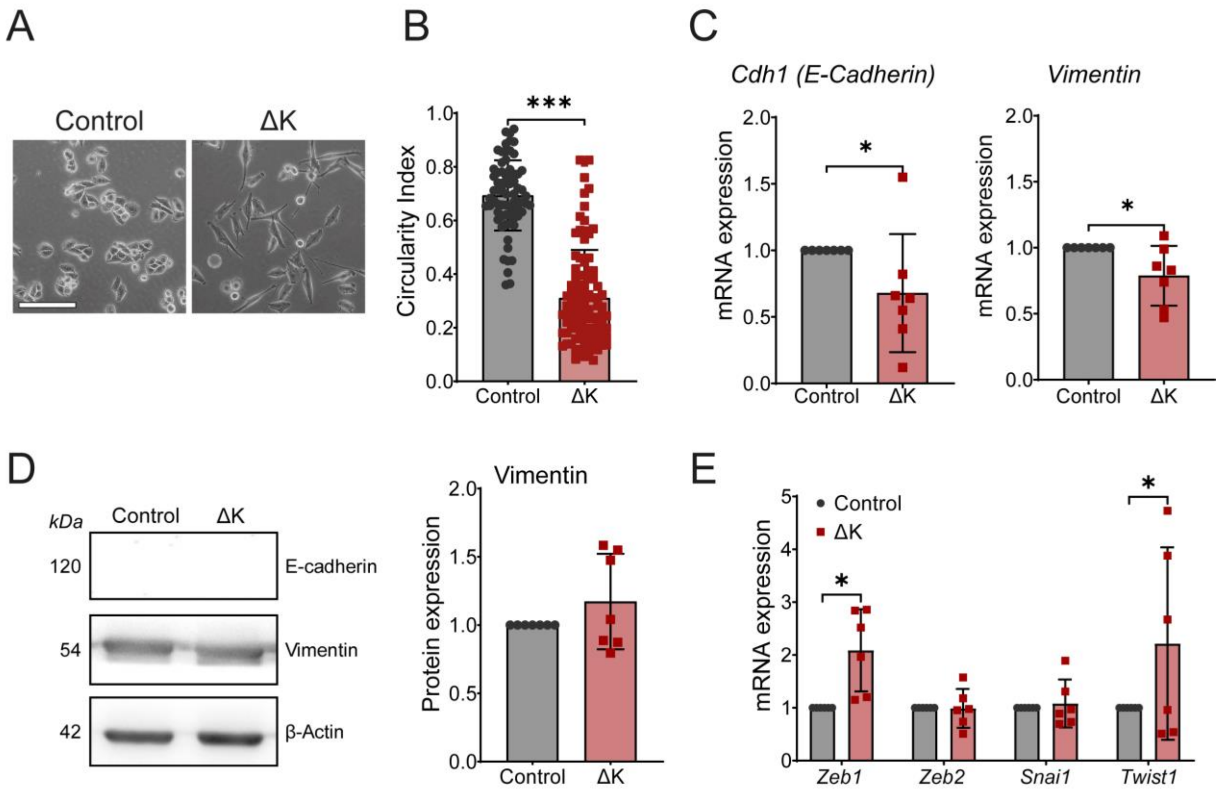

Figure S3: Effects of kinase domain deletion on EMT in MIA PaCa-2 cells.

A Representative morphology of MIA PaCa-2 Control and ΔK cells at x100 magnification. Scale bar=200µm. **B** Circularity index calculated for MIA PaCa-2 Control (n=71) and ΔK (n=98) cells (p<0.001, Mann-Whitney test). **C** mRNA expression of *CDH1* (n=7, p<0.01, Mann-Whitney test) and Vimentin (n=7, p<0.001, Mann-Whitney test) in MIA PaCa-2 Control and ΔK cells. **D** Protein expression of E-cadherin (n=7) and Vimentin (n=7 p>0.05, Mann-Whitney test) in MIA PaCa-2 Control and ΔK cells. E mRNA expression of EMT transcription factors *Zeb1*, *Zeb2*, *Snai1* and *Twist1* (n=6, p<0.05, Two-way ANOVA followed by Šidák's *post-hoc* test) in MIA PaCa-2 Control and ΔK cells. Results are shown as mean±SD. *p<0.05; **p<0.01; ***p<0.001.

Figure S4:

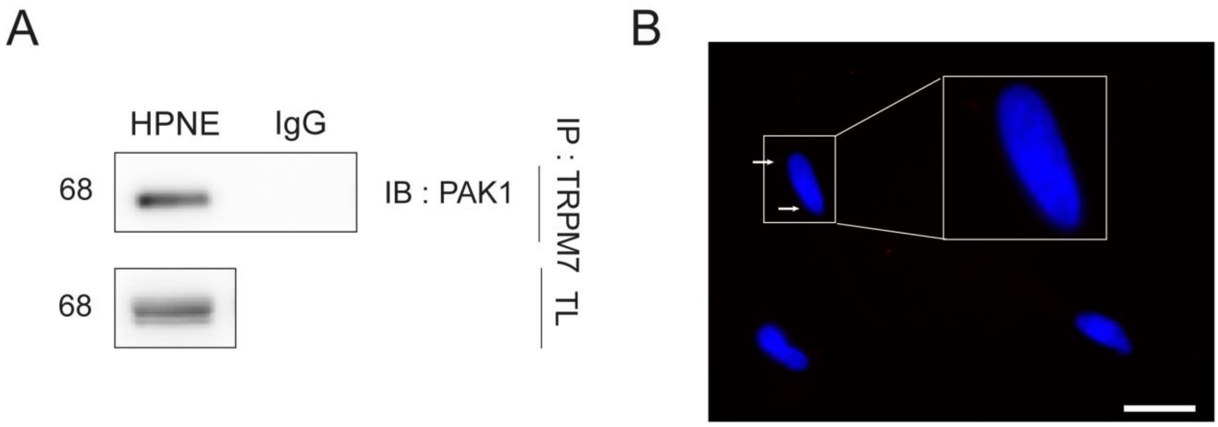

Figure S4: Interaction between TRPM7 and PAK1 in non-cancer cell line. **A** Co-immunoprecipitation of TRPM7 with PAK1 in normal pancreatic hTERT-HPNE cell line (n=3)

and its negative IgG control. **B** Proximity Ligation assay of TRPM7 with PAK1 in hTERT-HPNE cells. Scale bar = 20 µm.

Figure S5:

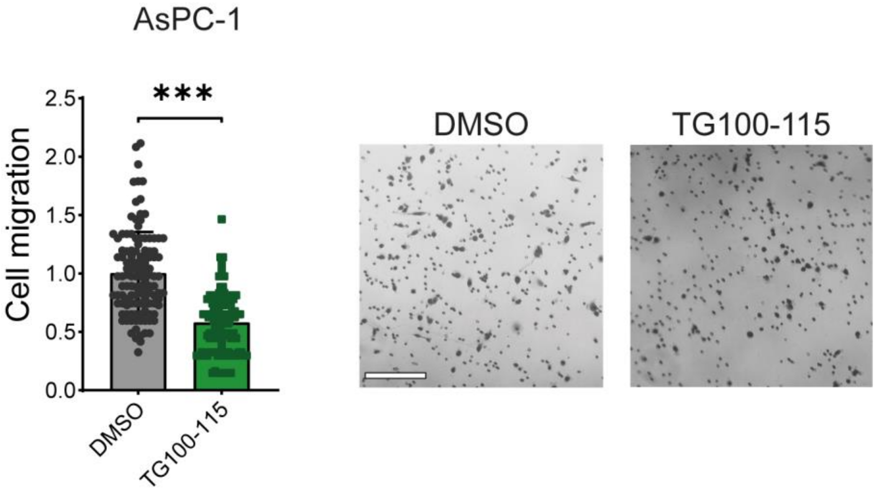

Figure S5: Effect of TG100-115 on KRASG12D mutated AsPC-1 cell migration. **A** Cell migration of AsPC-1 treated with 50 µM TG100-115 evaluated in Boyden chambers (n=3, p<0.001, Mann-Whitney test). Scale bar is corresponding to 200 µm. Results are shown as mean±SD. ***p<0.001.

Figure S6:

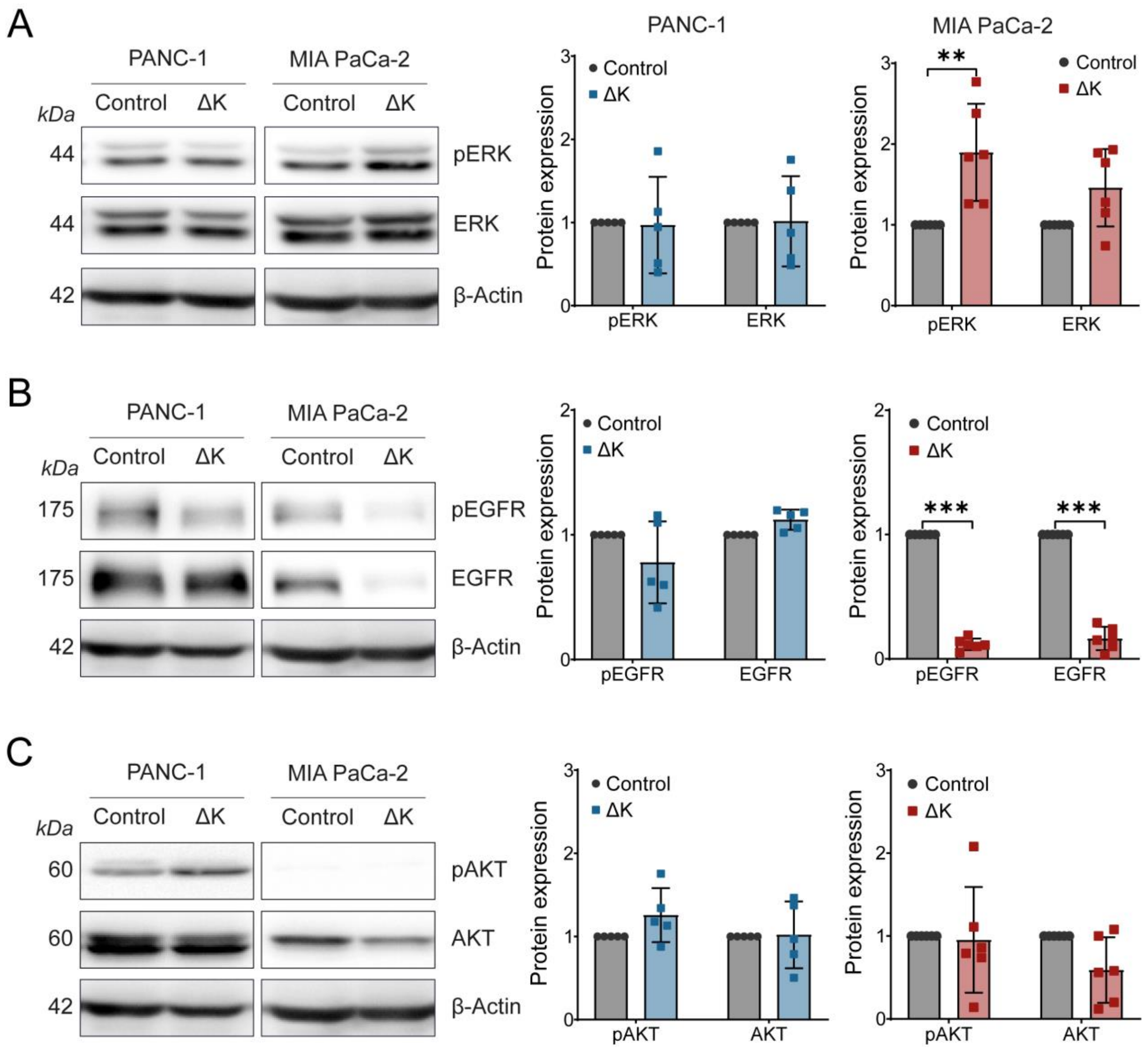

Figure S6: Effects of TRPM7 kinase domain deletion on ERK, AKT, and EGFR phosphorylation in PANC-1 and MIA PaCa-2 cell lines. **A** Expression and phosphorylation levels of ERK protein in PANC-1 (n=5, Two-way ANOVA followed by Šidák's *post-hoc* test) and MIA PaCa-2 cells (n=6, p<0.01, Two-way ANOVA followed by Šidák's *post-hoc* test). **B** Expression and phosphorylation levels of EGFR protein in PANC-1 (n=5, Two-way ANOVA followed by Šidák's *post-hoc* test) and MIA PaCa-2 cells (n=6, Two-way ANOVA followed by Šidák's *post-hoc* test) **C**. Expression and phosphorylation levels of AKT protein in PANC-1 (n=5, Two-way ANOVA followed by Šidák's *post-hoc* test) and MIA PaCa-2 cells (n=6, p<0.001, Two-way ANOVA followed by Šidák's *post-hoc* test). Results are shown as mean±SD. *p<0.05; **p<0.01; ***p<0.001.

The original western-blots including full length uncropped original data are available as supplemental material.
