# Supplementary material for: The kinase domain of TRPM7 interacts with PAK1 and regulates pancreatic cancer cell epithelial-to-mesenchymal transition
Source: Cell Death Dis. 2025 Apr 24;16(1):335. doi: 10.1038/s41419-025-07665-2 (PMC12022261; doi:10.1038/s41419-025-07665-2)

Uncropped Western-Blots – Figure 1C

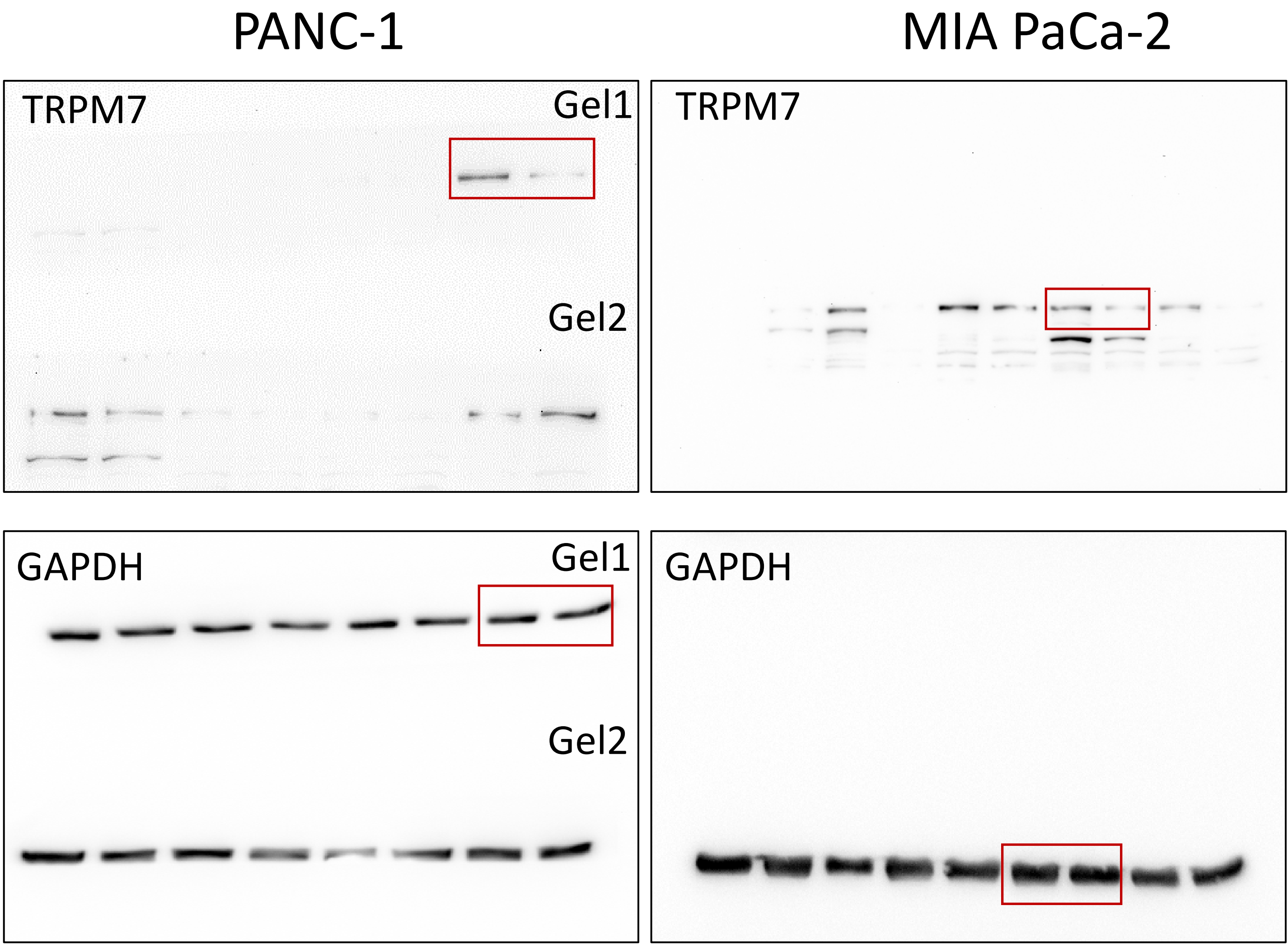

Uncropped Western-Blots – Figure 3D

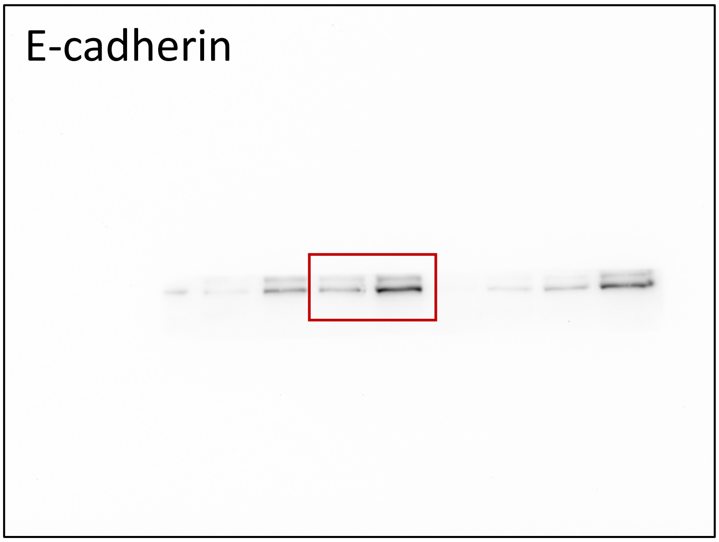

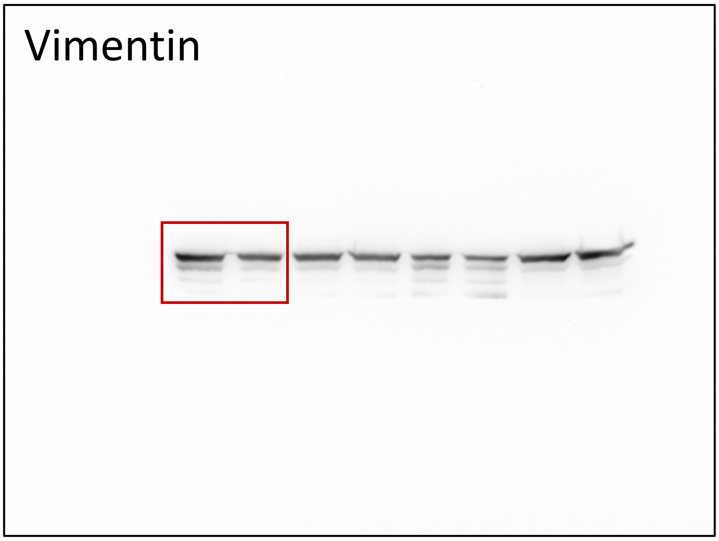

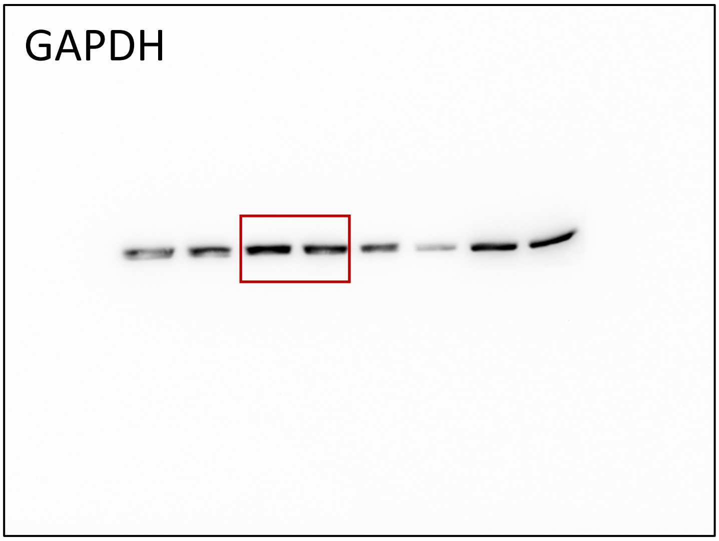

Uncropped Western-Blots – Figure 5A

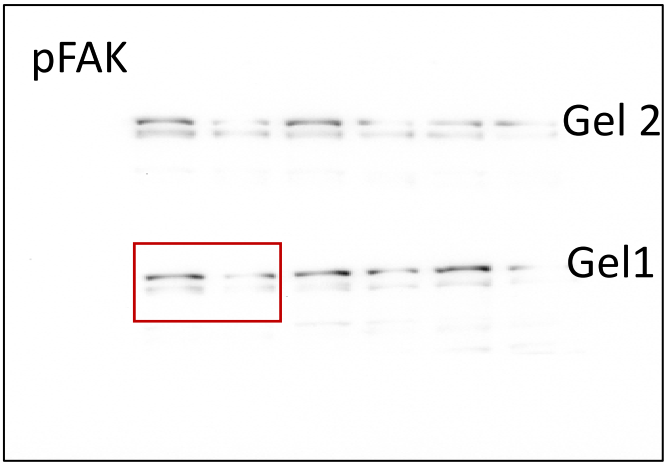

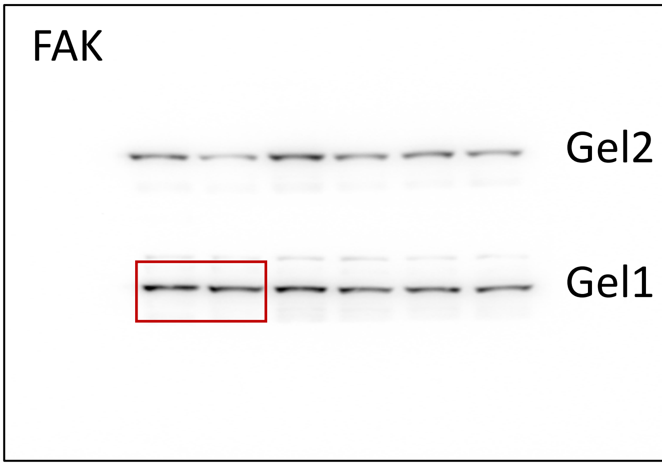

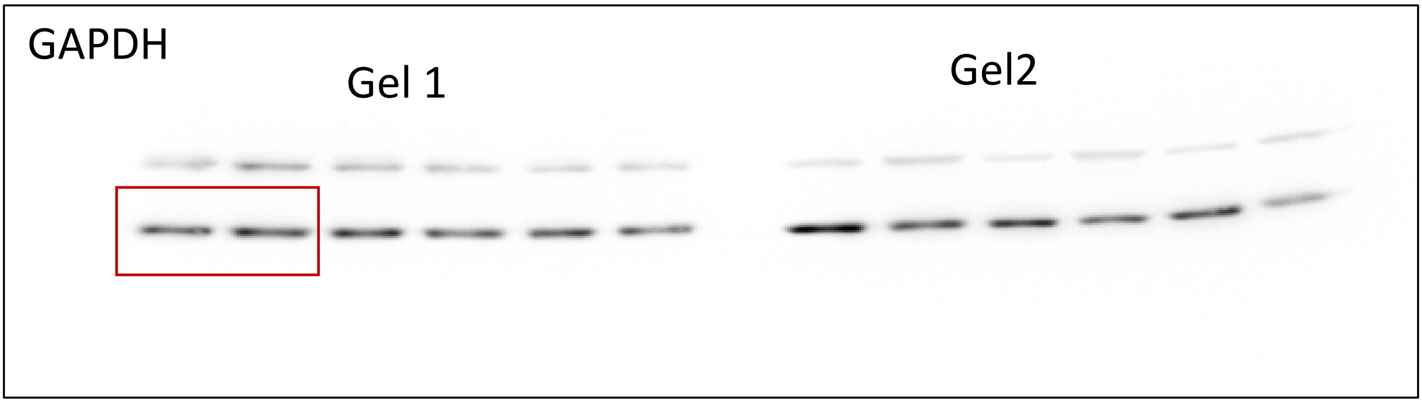

Uncropped Western-Blots – Figure 5B

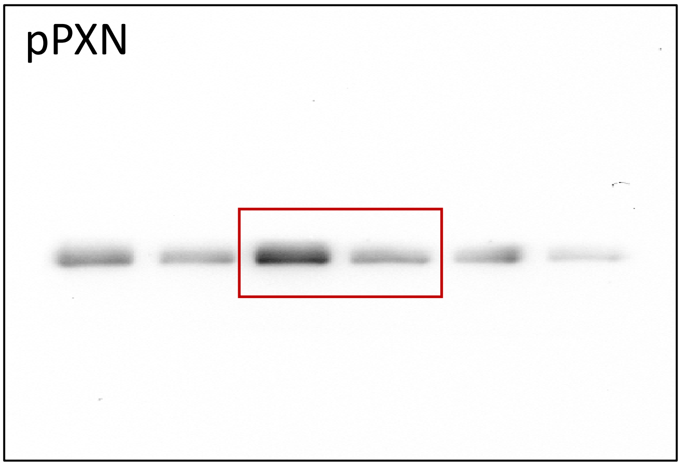

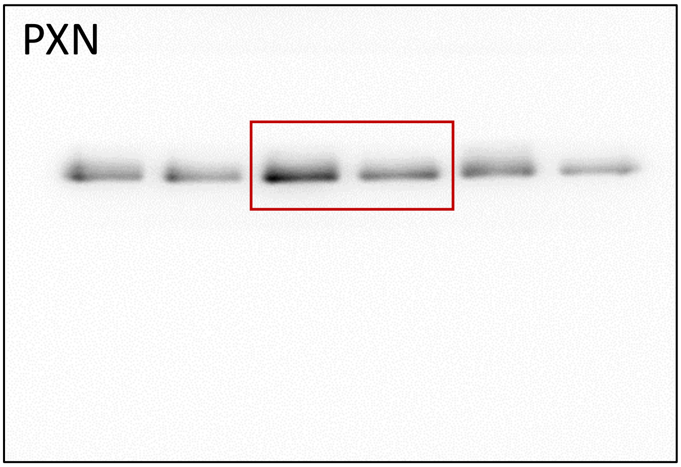

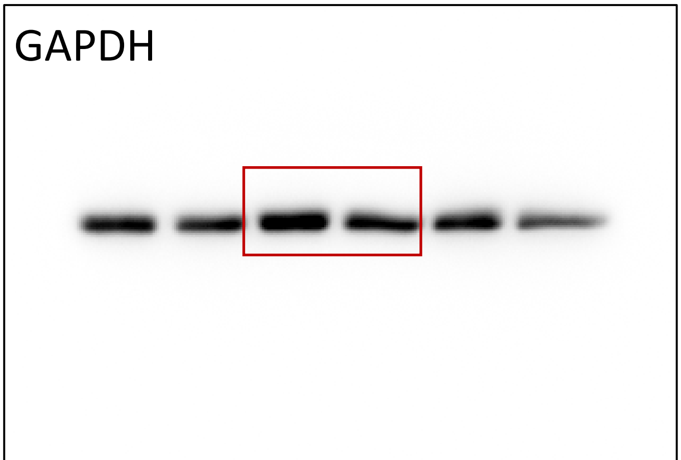

Uncropped Western-Blots – Figure 5C

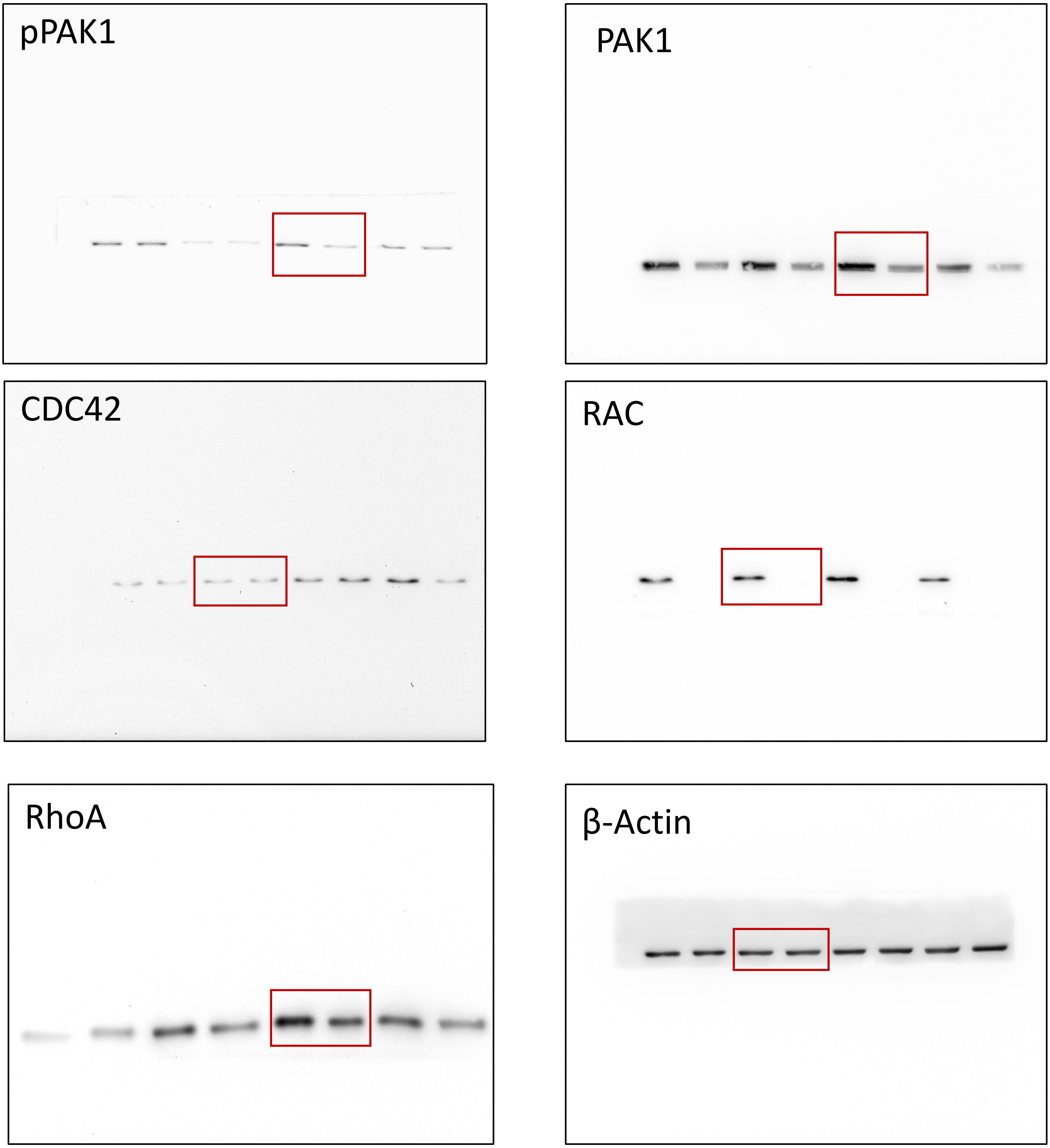

Uncropped Western-Blots – Figure 5D

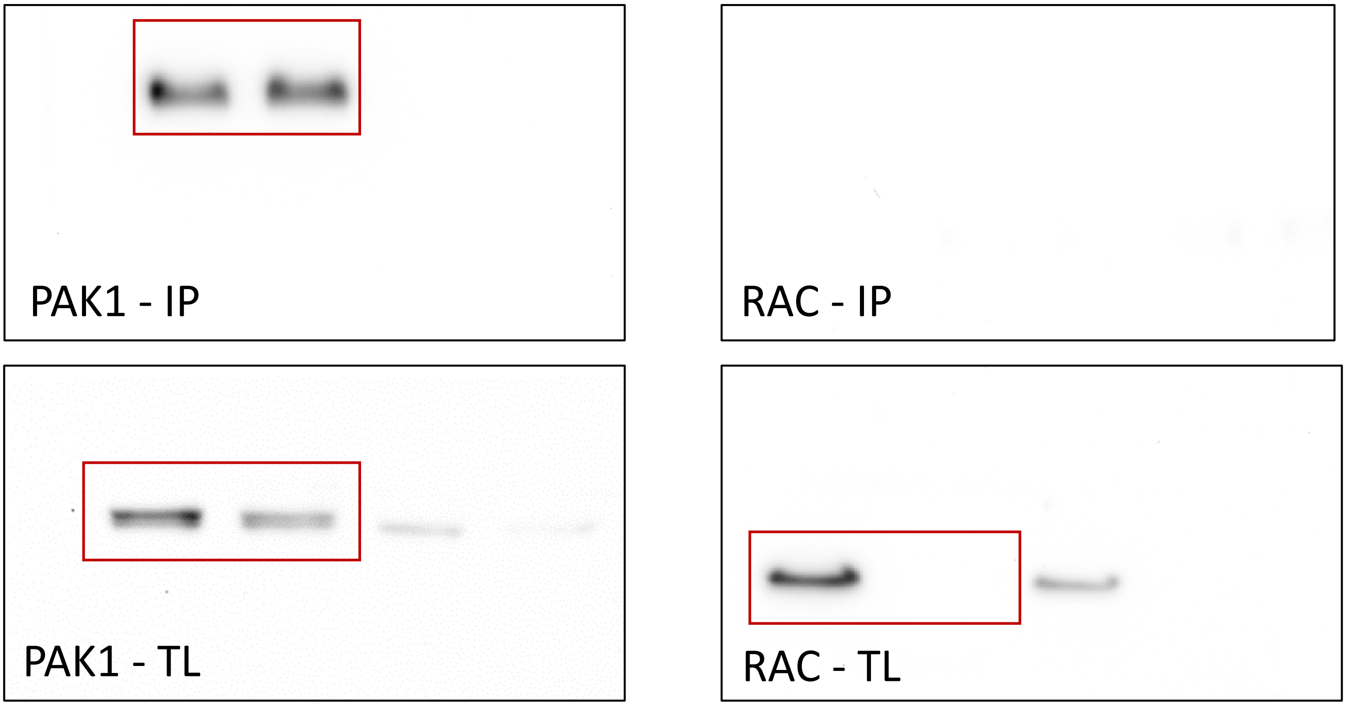

Uncropped Western-Blots – Figure 7E

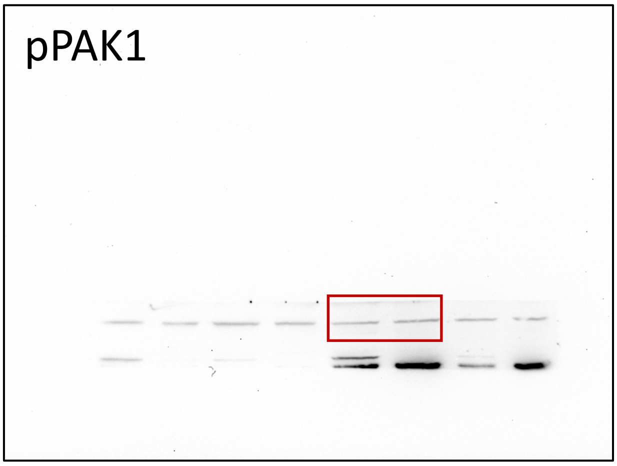

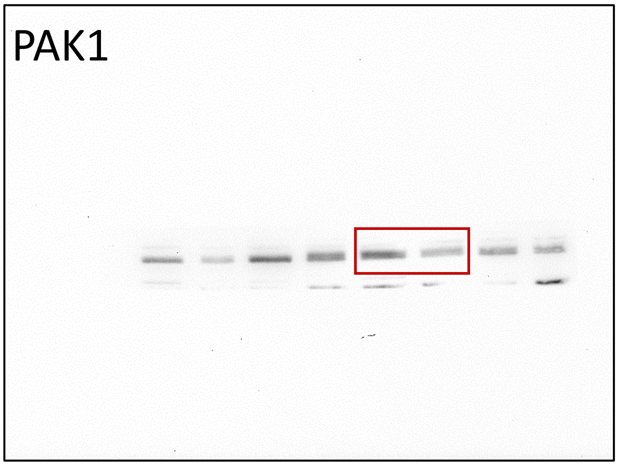

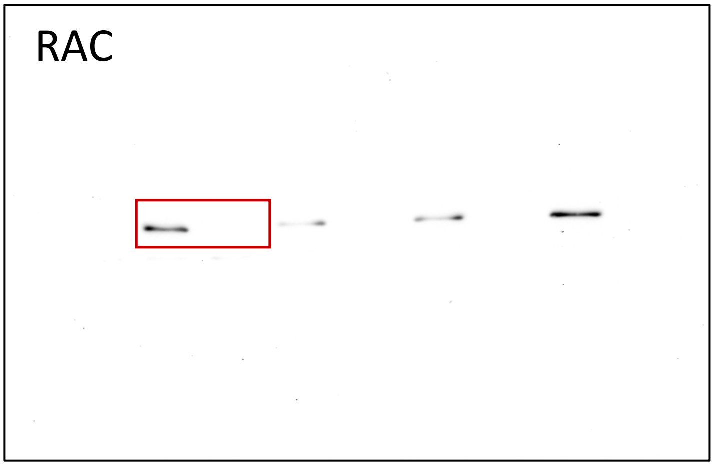

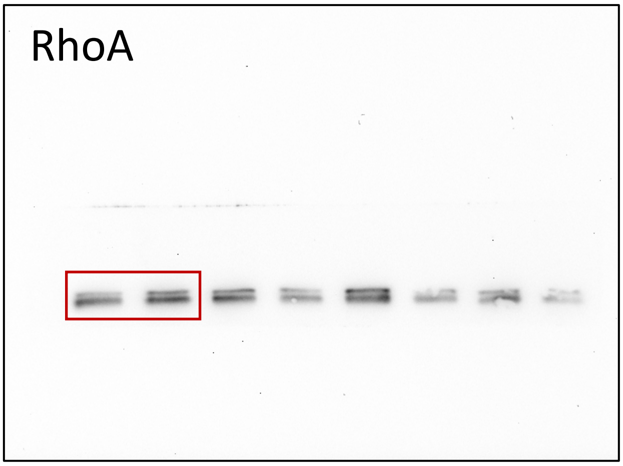

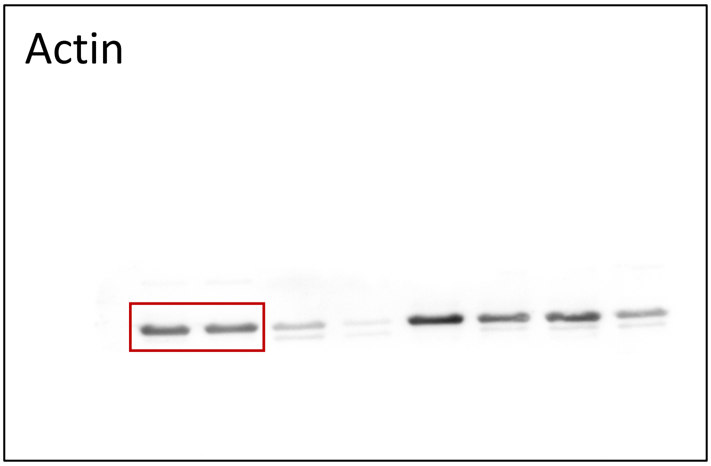

Uncropped Western-Blots – Figure 7F

PAK1 - IB

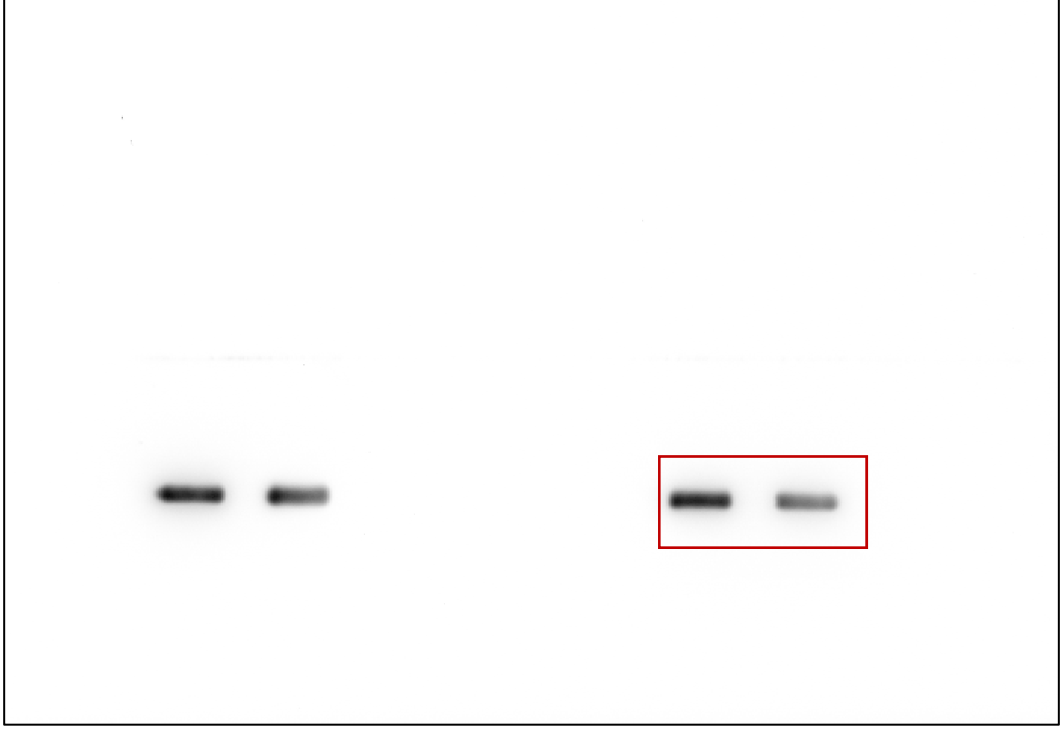

PAK1 - TL

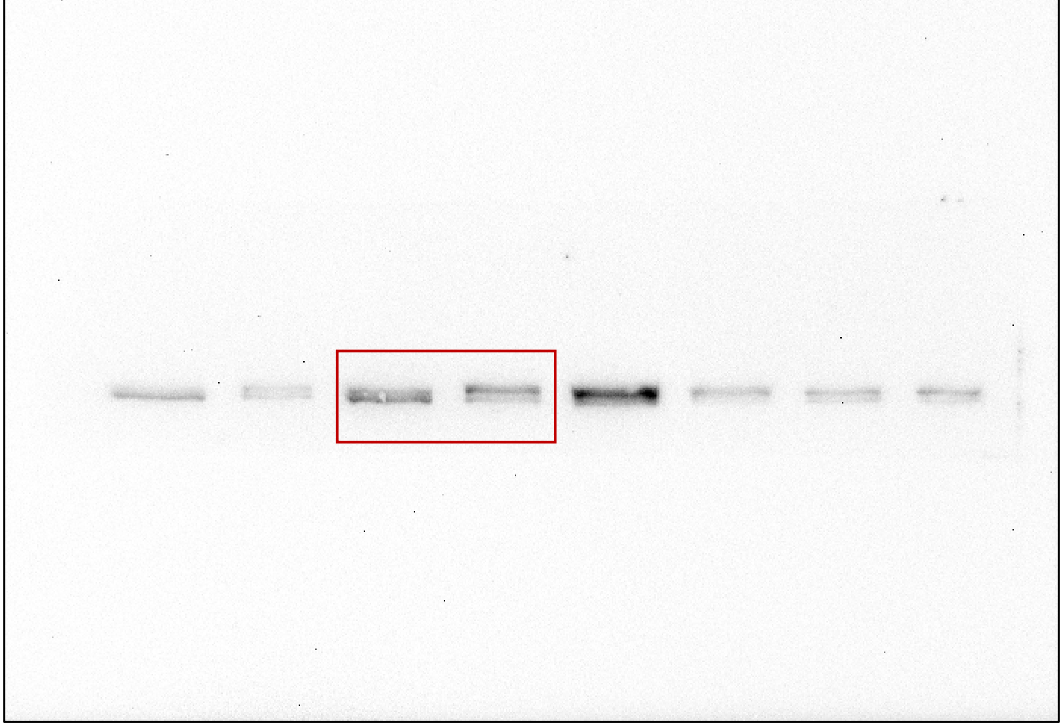

Uncropped Western-Blots – Figure 7J (1/2)

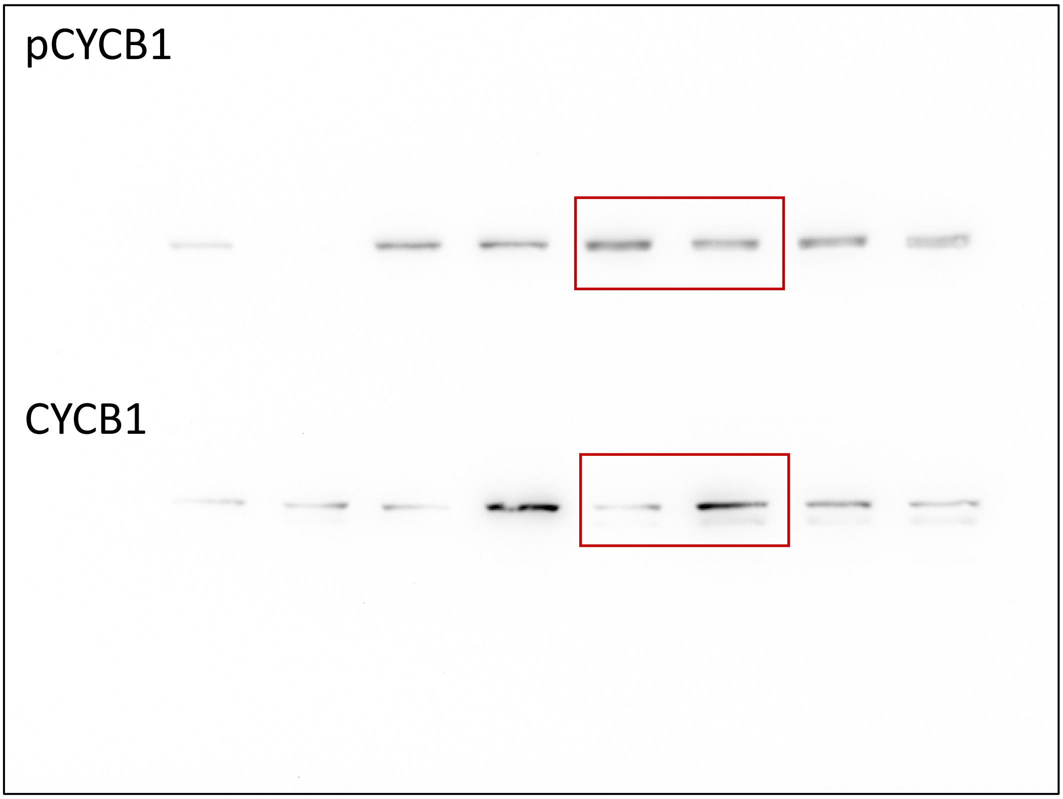

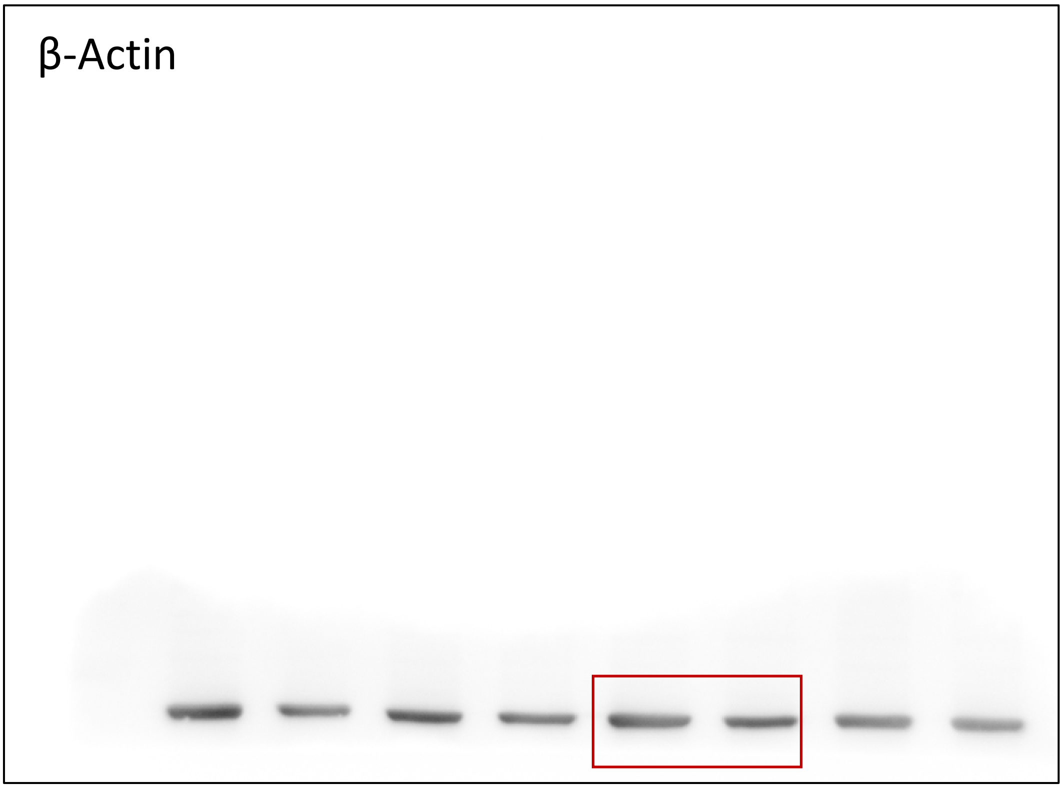

Uncropped Western-Blots – Figure 7J (2/2)

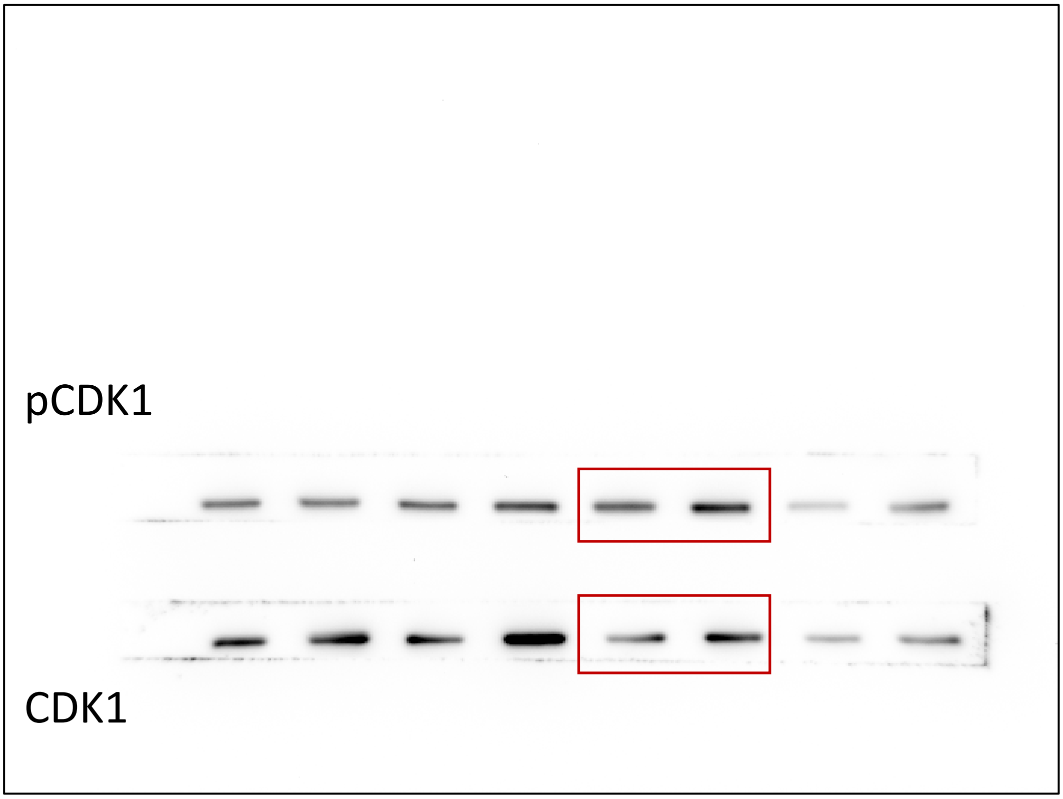

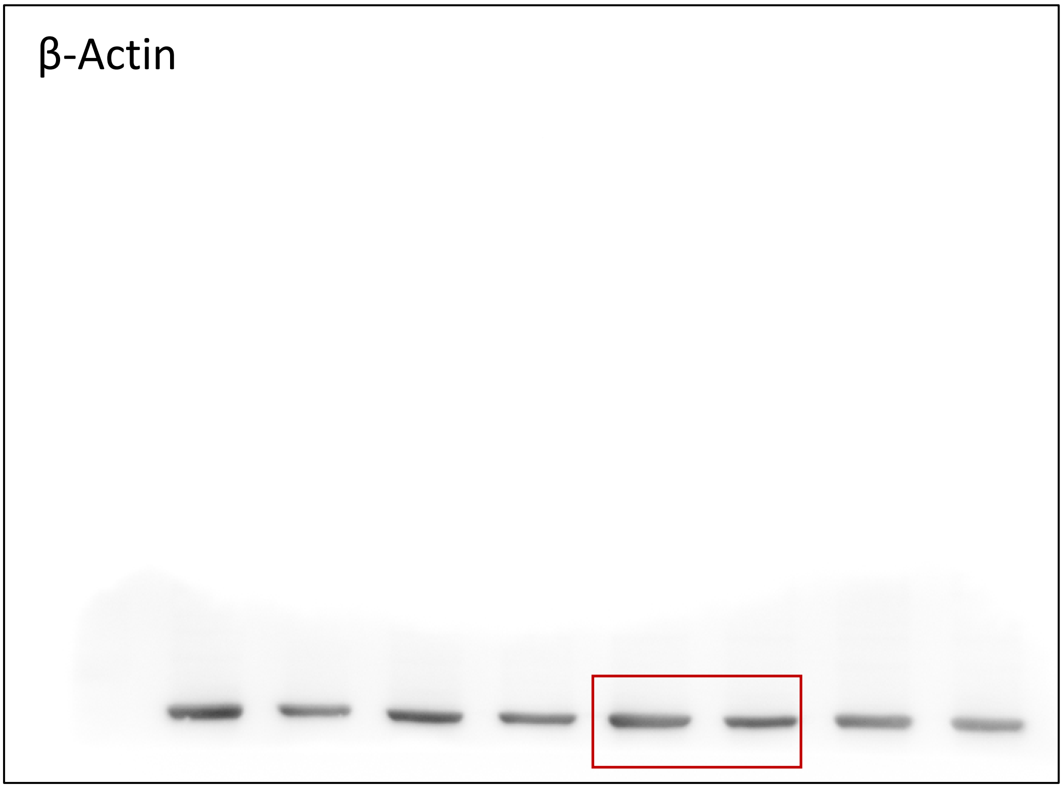

Uncropped Western-Blots – Supplementary figure S1

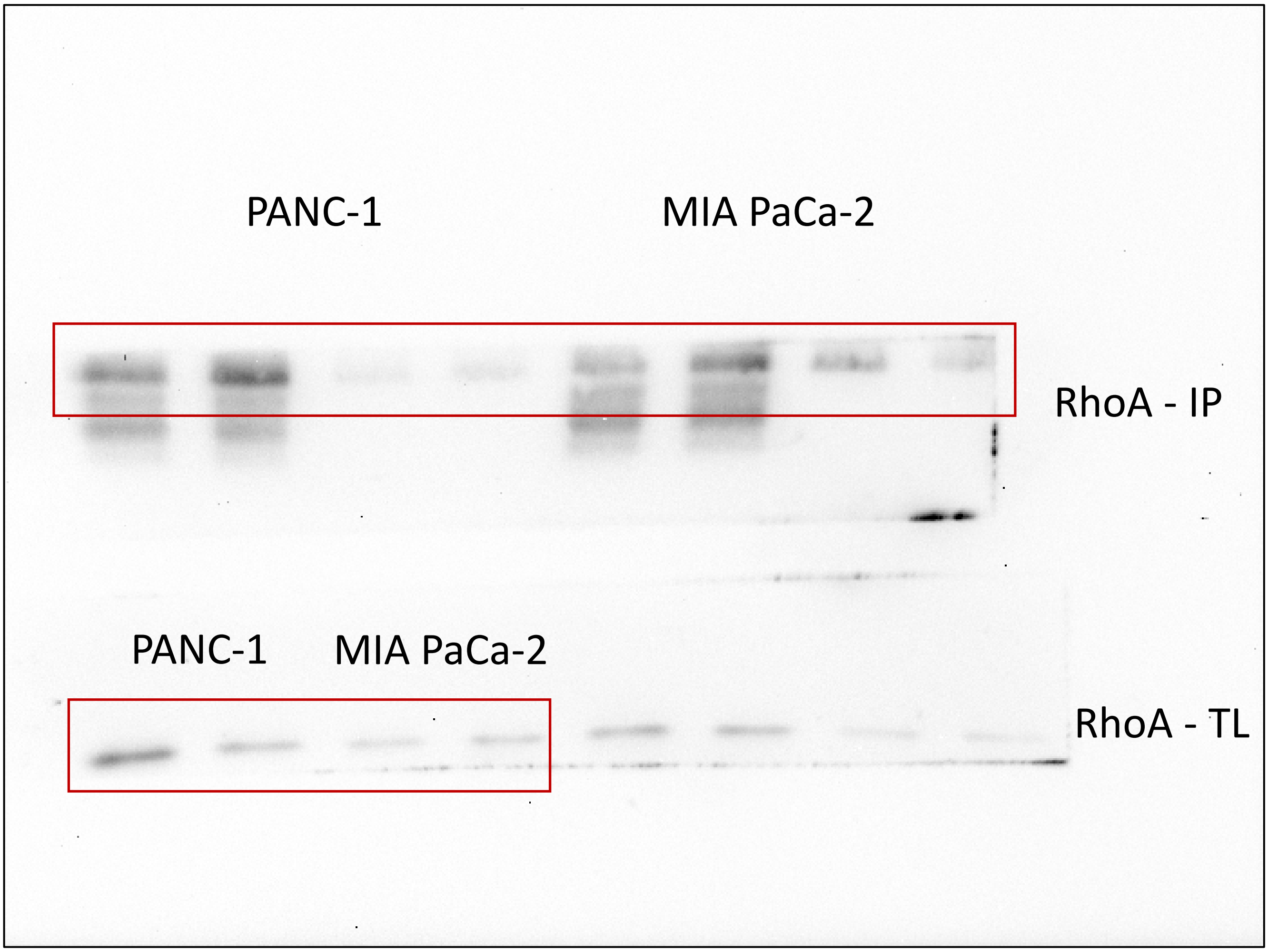

Uncropped Western-Blots – Supplementary figure S3A

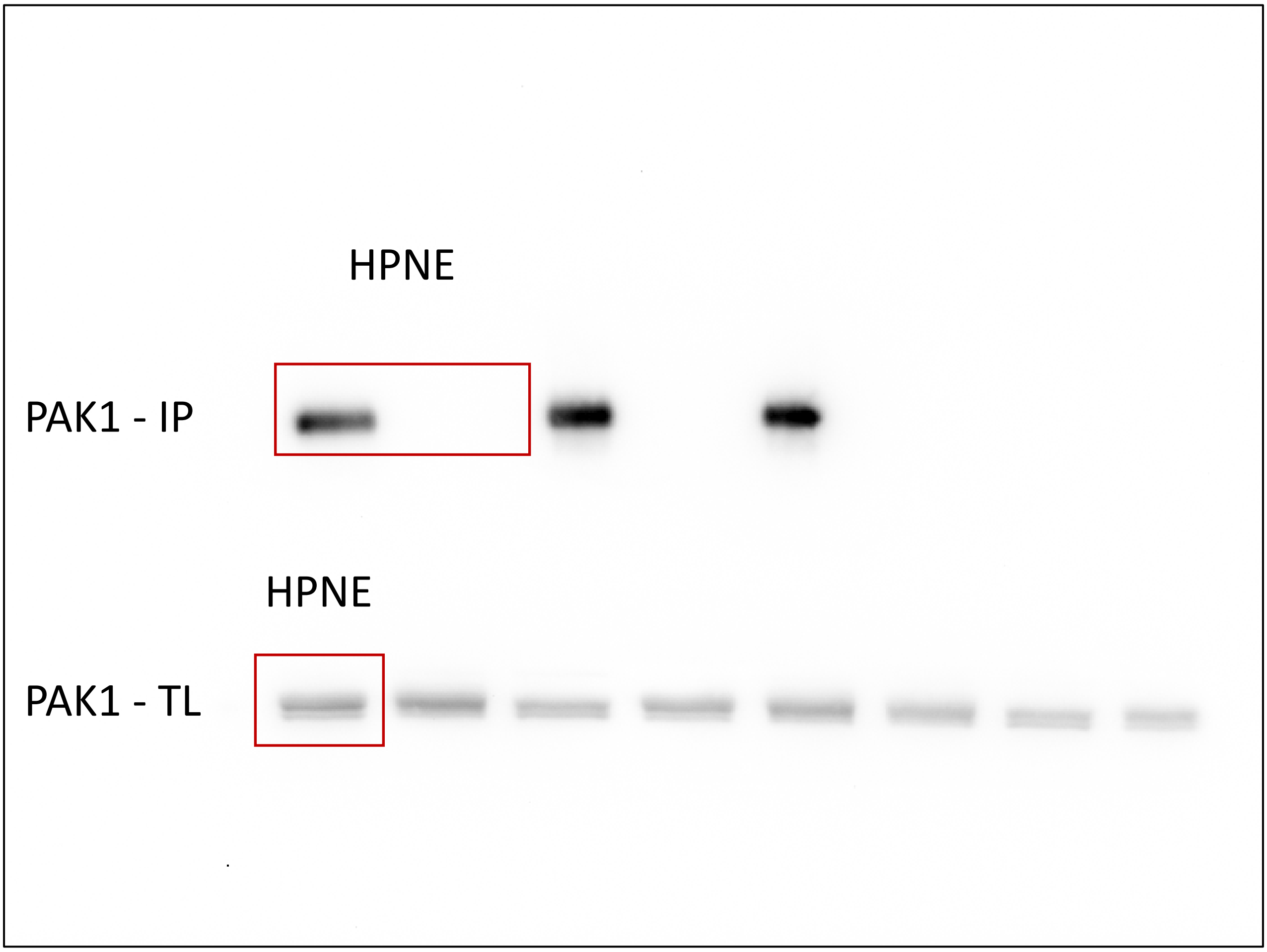

Uncropped Western-Blots – Supplementary figure S5A

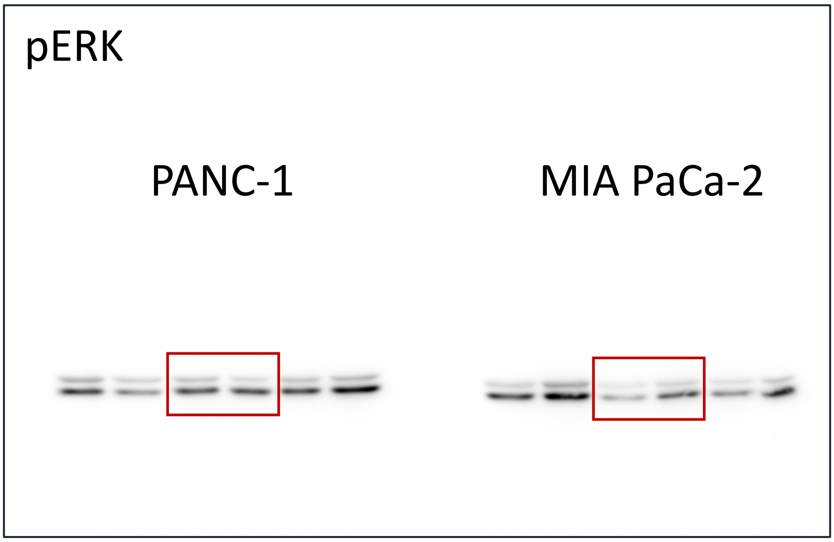

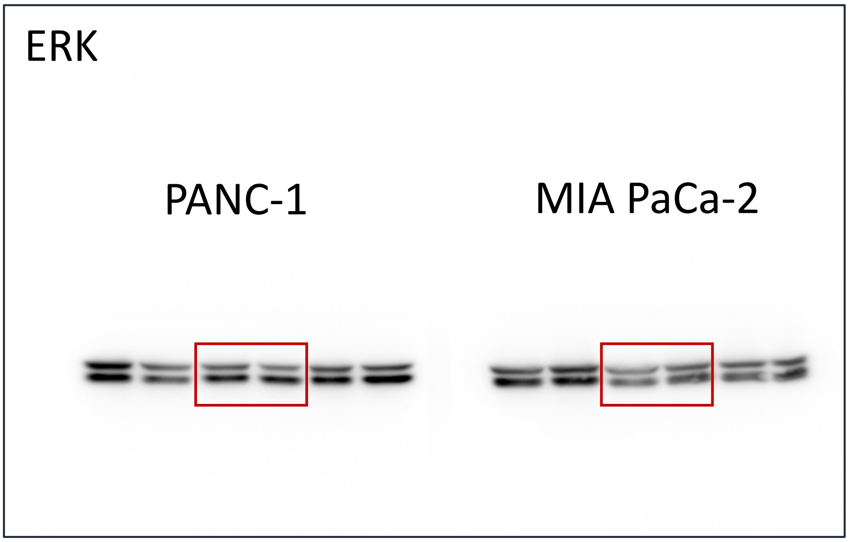

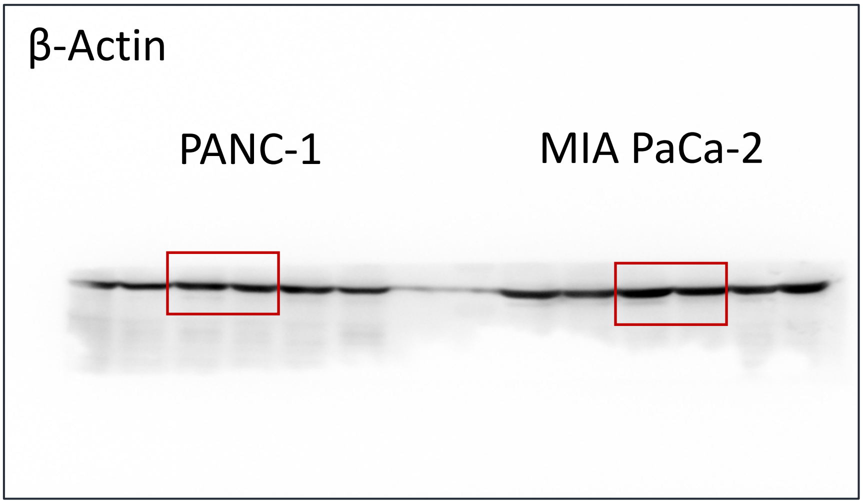

Uncropped Western-Blots – Supplementary figure S5B

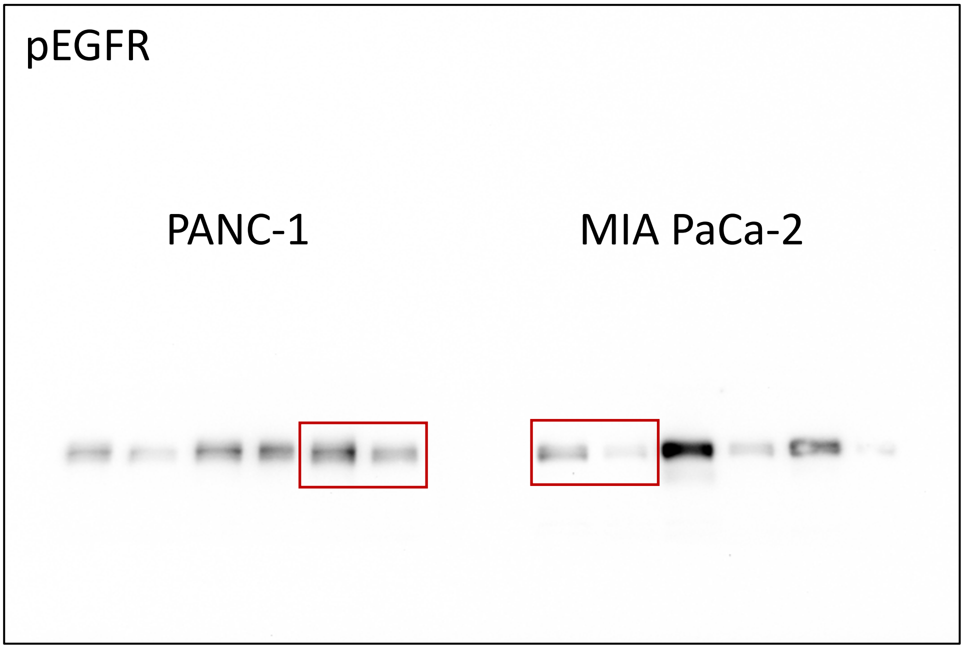

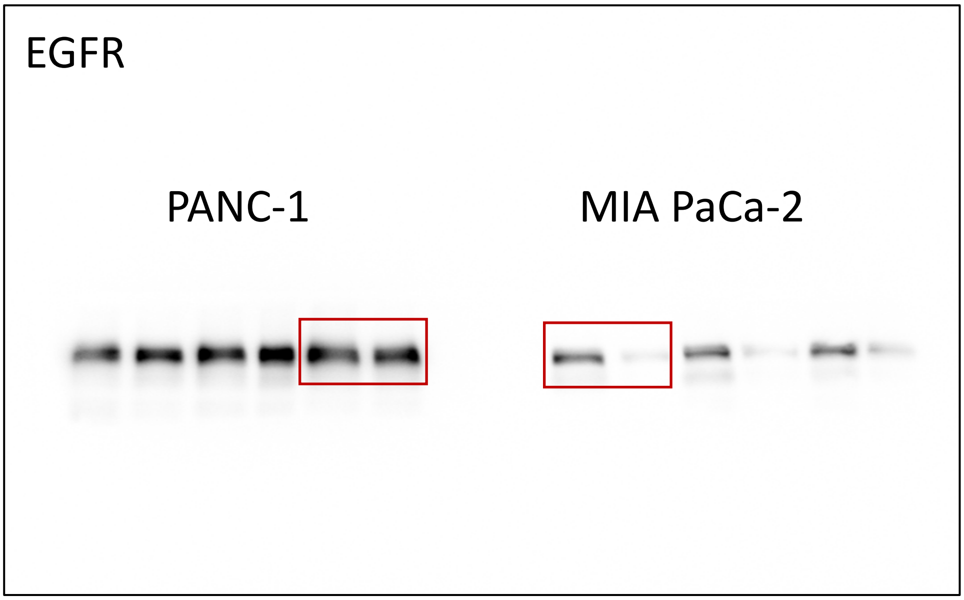

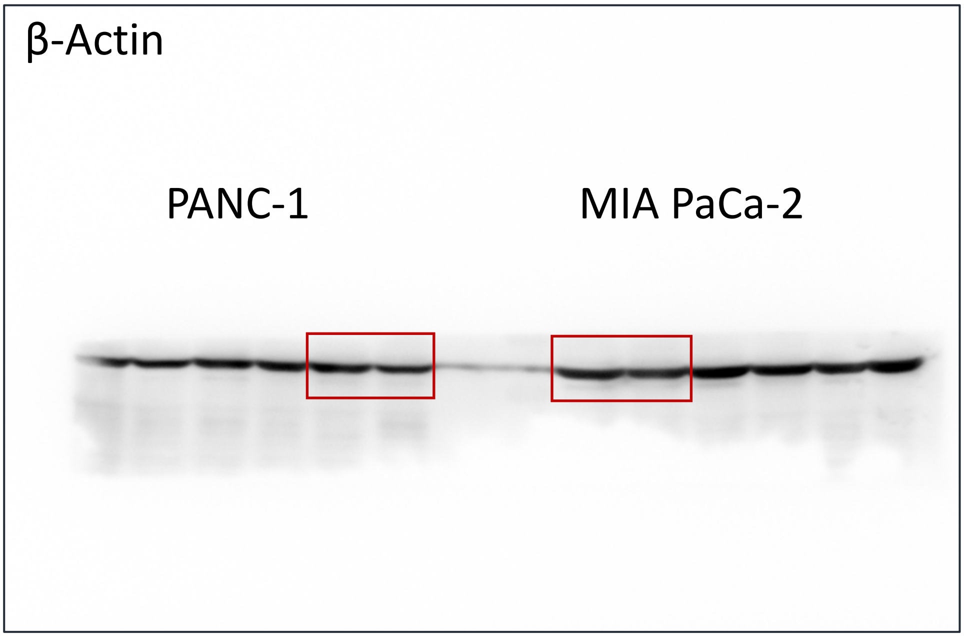

Uncropped Western-Blots – Supplementary figure S5C

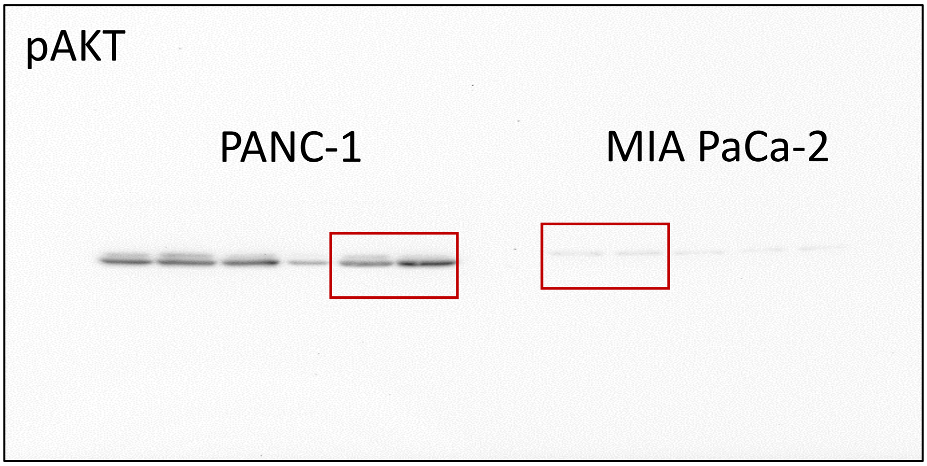

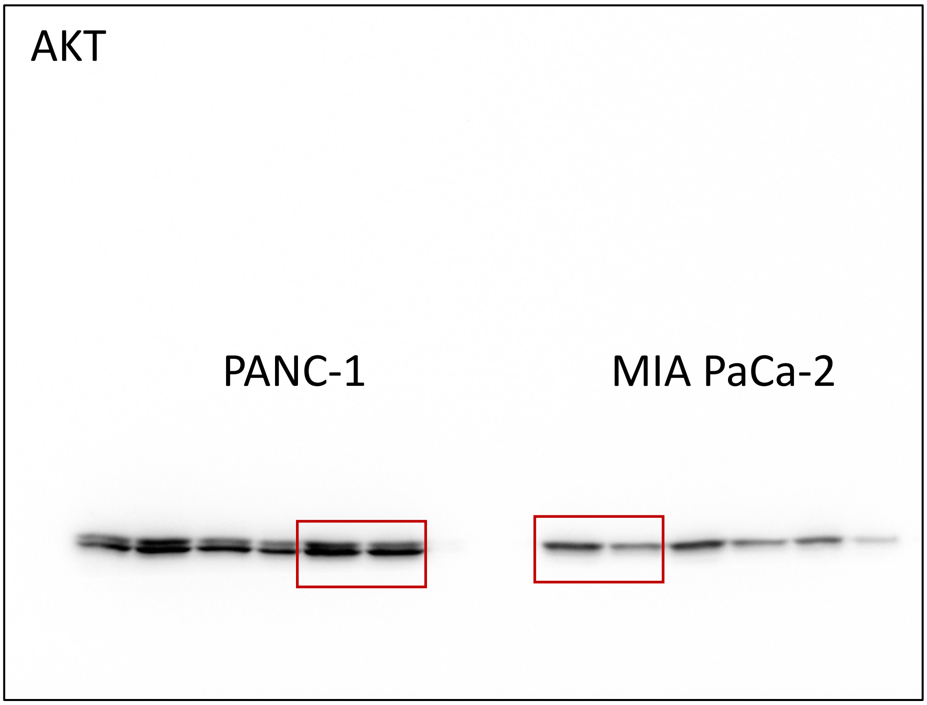

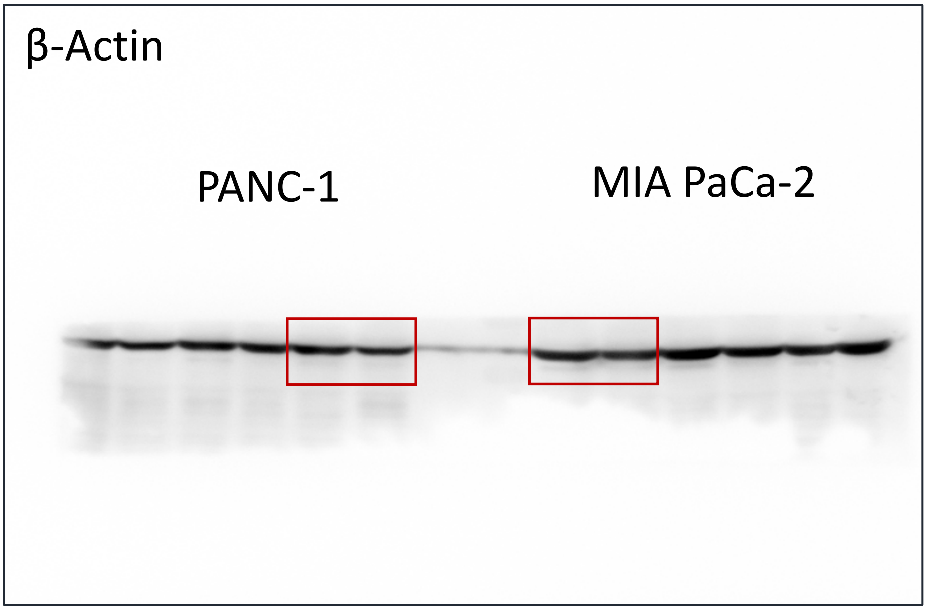

Supplement: Supplementary file 2 — Original western blots [file 41419_2025_7665_MOESM2_ESM.pdf]
